# Neuroprotective Effect of CR-777, a Glutathione Derivative of Withaferin A, Obtained through the Bioconversion of *Withania somnifera* (L.) Dunal Extract by the Fungus *Beauveria bassiana*

**DOI:** 10.3390/molecules24244599

**Published:** 2019-12-16

**Authors:** Chérif Rabhi, Guillaume Arcile, Géraldine Le Goff, Christian Da Costa Noble, Jamal Ouazzani

**Affiliations:** 1 Laboratoire Ethnodyne, 151 Boulevard Haussmann, 75008 Paris, France; c.rabhi@ethnodyne.com (C.R.); cdcn@horus-finance.com (C.D.C.N.); 2 Institut de Chimie des Substances Naturelles ICSN, Centre National de la Recherche Scientifique, Avenue de la Terrasse, 91198 Gif-sur-Yvette, France; guillaume.arcile@cnrs.fr (G.A.); geraldine.legoff@cnrs.fr (G.L.G.)

**Keywords:** *Withania somnifera*, bioconversion, withaferin A, glutathione conjugate, Alzheimer’s disease, Parkinson’s disease, neuroprotection

## Abstract

The bioconversion of *Withania somnifera* extract by the fungus *Beauveria bassiana* leads to cysteine and glutathione derivatives of withaferin A at the C-6 position. The compounds were purified and fully characterized by 1D-NMR, 2D-NMR, and HRMS analysis. The glutathione derivative CR-777 was evaluated as a neuroprotective agent from damage caused by different neurotoxins mimicking molecular symptoms in Parkinson´s disease (PD), including 1-methyl-4-phenylpyridinium (MPP+), 6-hydroxydopamine (6-OHDA), and α-synuclein (α-Syn). CR-777, at nanomolar concentrations, protected dopaminergic and cortical neurons. In 6-OHDA-treated neurons, CR-777 increased cell survival and neurite network and decreased the expression of α-Syn. Using specific inhibitors of cell toxicity signaling pathways and specific staining experiments, the observed role of CR-777 seemed to involve the PI3K/mTOR pathway. CR-777 could be considered as a protective agent against a large panel of neuronal stressors and was engaged in further therapeutic development steps.

## 1. Introduction

Neurodegenerative diseases include different pathologies such as Alzheimer’s disease (AD) and Parkinson’s disease (PD). The common feature in these diseases is the progressive loss or degeneration of dopaminergic neurons, leading to cognitive and motor disorders. The neuron degeneration is multifactorial, including environmental, molecular, cellular, and genetic factors, and involves an increase in oxidative stress, impaired cell signaling and functions, deposition of aggregated proteins, and inflammation.

Herbal ingredients and their constituents were extensively investigated for the prevention and treatment of AD [1] and PD [2,3]. Among them, *Withania somnifera* (Ashwagandha) is receiving attention due to its diverse and specific chemical composition [4,5]. Beyond neuroprotection [6,7], many activities were reported for *Withania somnifera* extracts or pure compounds, including antibacterial, anticancer, antidiabetic, cardioprotective, and anti-inflammatory properties [5]. Several patents on different compositions of plant extracts have been filed [8]; however, no single pharmacological compound has been developed to date.

Since many side and adverse effects are suspected for *Whitania* extracts, and considering the widespread use of the plant in traditional medicine, extensive studies were undertaken to regulate the traditional use of the plant. The authors of these studies concluded that the plant extract is devoid of acute or sub-acute toxicity [9,10]. According to the European Medicines Agency (EMA) and the Committee on Herbal Medicinal Products (HMPC), in a public statement on *Withania somnifera* (L.) in 2013 it was concluded that an assessment of efficacy and safety should be completed (EMA/HMPC/681519, 2012). A new evaluation by HMPC was conducted in 2018, and the final assessment has not been communicated yet.

In order to rationalize therapeutic benefits and accurately investigate side adverse effects, the development of a single bioactive molecule represents the appropriate alternative.

**Figure 1:**
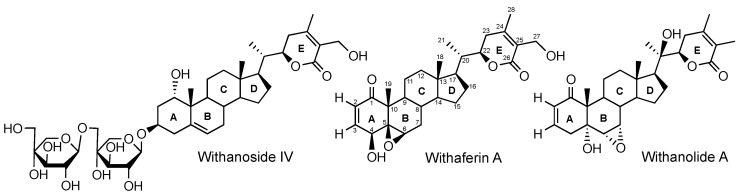
Examples of the withanolide and withanoside scaffolds of *Withania somnifera* constituents.

Among the *Withania somnifera* constituents, withaferin A has attracted considerable attention due its wide range of multifunctional bioactivities [11,12,13,14]. However, in neurodifferentiation, neuroprotection, and neuroregeneration, withaferin A is not the most active constituent compared to withanolide A and withanoside IV (Figure 1) [4].

Due to its toxicity, different withaferin A analogs were synthetized; among them the 3-azido derivative [15] and the oxidized forms of the epoxide [16] were reported to be more cytotoxic than the parent withaferin A. The apoptotic activity of withaferin A can be also modulated according to structure-based design of different analogs [17].

Biocatalysis is the conversion of compounds or mixture of compounds using living microbial cells or enzymes. It is a biocompatible green alternative to chemical reactions [18,19]. Emerging evidence suggests that biocatalysis enhances the bioactivity and therapeutic potential of traditional medicines [20,21,22,23].

The fungus *Beauveria bassiana* (Ascomycota, Cordycipitaceae), is an entomopathogen used for microbial control of pests and for the elicitation of plant defense against microbial invaders [24]. This fungus is also widely used in biocatalysis for the unique diversity and effectiveness of the catalyzed reactions [25].

We previously reported on a combination of three Ayurvedic medicinal plant extracts (*Withania somnifera*, *Emblica officinalis*, *Bacopa monnieri*) submitted to fermentation with the fungus *Beauveria bassiana.* The fermented mixture was screened in cellulo and in ovo and exhibits beneficial angiogenic and neuro-protective properties [26,27]. The non-fermented mixture was not active, but chowed significant cell toxicity. Individual extract investigation demonstrated that most of the activity is associated to the fermented extract of *Withania somnifera* [28,29]. In an attempt to isolate and identify the active molecules in the fermented *Withania somnifera* extract, two withaferin derivatives were isolated, fully characterized, and showed neuroprotective activity.

In this paper we reported the isolation and structural elucidation of two withaferin A conjugates: the cysteine derivative CR-591 (**1**) and the glutathione derivative CR-777 (**2**). The latter protects dopaminergic and cortical neurons against PD mimicking injuries.

## 2. Results

### 2.1. Production, Isolation and Structural Elucidation of the Bioconversion Products Produced through the Bioconversion of *Withania somnifera* Extract by *Beauveria bassiana* ATCC 7159

**Figure 2:**
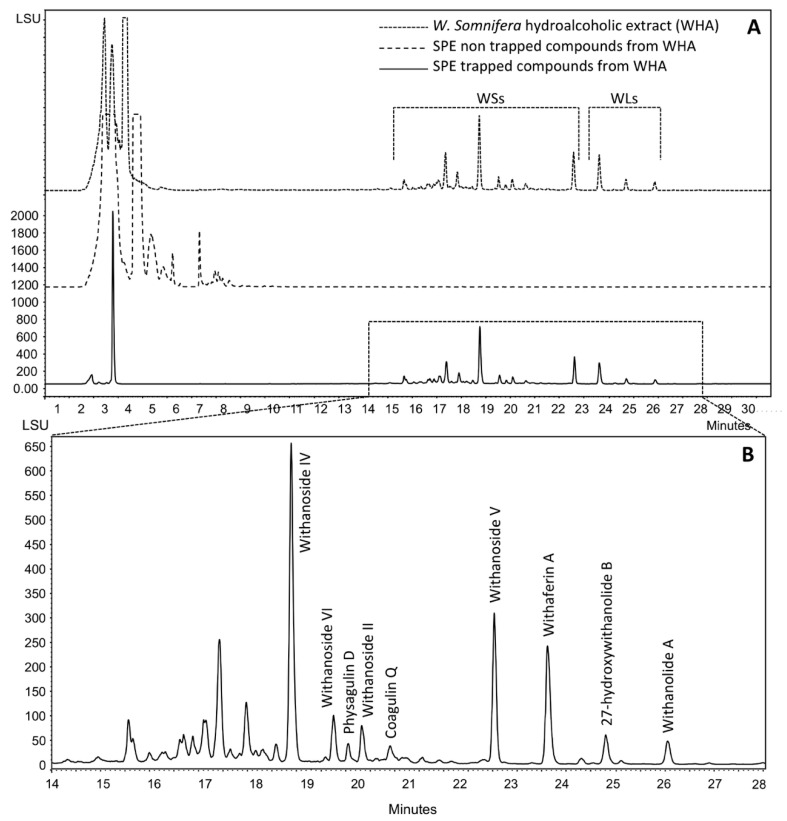
HPLC analysis of different *Withania somnifera* extracts (**A**) and withanoside/withanolide mixture (**B**). SPE: solid-phase extraction; WSs: withanosides; WLs: withanolides.

The hydroalcoholic extract of *Withania somnifera* WHA was prepared as detailed in the experimental section. Figure 2 shows that besides the withanosides (WSs) and withanolides (WLs) eluted between 14 and 27 min, a mixture of polar compounds was eluted in the first 10 min of the chromatogram. These compounds, accounting for 91% of the whole hydroalcoholic extract, were easily removed after trapping of target WSs and WLs by solid-phase extraction (SPE) on Amberlite XAD-1600N resin. Target compounds were desorbed from the resin by methanol and recovered by evaporation offering 9% of the whole hydroalcoholic extract; this mixture is referred to as WE-SPE.

WE-SPE was submitted to resting cells fermentation with *Beauveria bassiana* ATCC 7159 as reported in the experimental section. Samples were recovered daily and analyzed by HPLC. After 5 days of incubation, the HPLC profile remained unchanged and the medium was filtered through a 0.2-μm membrane and dried by lyophilization.

**Figure 3:**
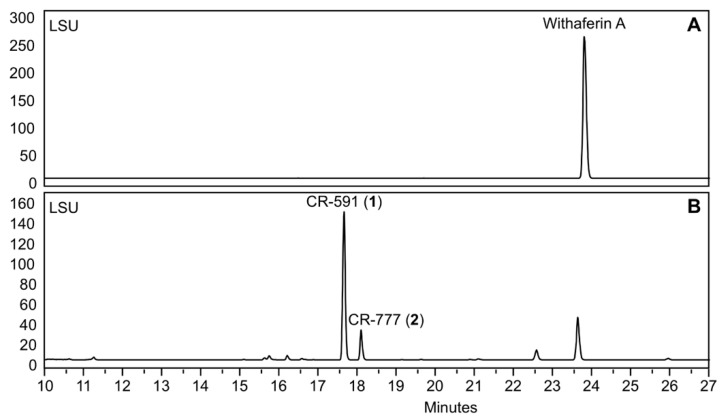
HPLC analysis during the incubation of withaferin A with *Beauveria bassiana* ATCC 7159. (**A**) Starting chromatogram, (**B**) after 5 days of incubation.

**Figure 4:**
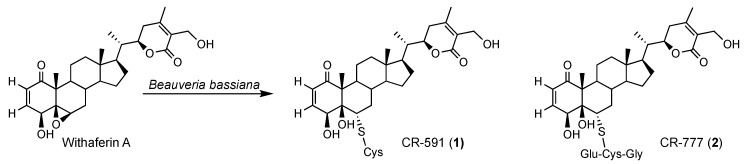
Structures of compounds **1** and **2** obtained from withaferin A during resting cell fermentation of *Withania somnifera* extract.

Besides different known compounds isolated by preparative HPLC and identified by NMR and HRMS-based dereplication (withanolide A, withanosides I to VI, physagulin D and coagulin Q), we characterized the unknown compound **1** with *m*/*z* 591 and **2** with *m*/*z* 777; both derive from withaferin A. In order to increase the yield of the two derivatives, withaferin A was isolated from WE-SPE and submitted to *Beauveria bassiana* ATCC 7159 biotransformation (Figure 3 and Figure 4).

Compound **1** was isolated as a white powder. Its molecular formula, C_31_H_45_NO_8_S, was deduced by HRESIMS with *m*/*z* 592.2953 [M + H]^+^. This formula was corroborated by the 13C NMR spectrum and revealed ten degrees of unsaturation. These degrees of unsaturation were attributed to the α,β-unsaturated δ-lactone moiety, the five-membered ring linked to three six-membered rings, one of which consists of a cyclohex-2-en-1-one and one carboxylic group. The IR spectrum exhibited a characteristic broad absorption band at 3363 cm^−1^ (OH), and a strong absorption band at 1670 cm^−1^ (α,β-unsaturated δ-lactone, and carbonyl groups).

**Figure 5:**
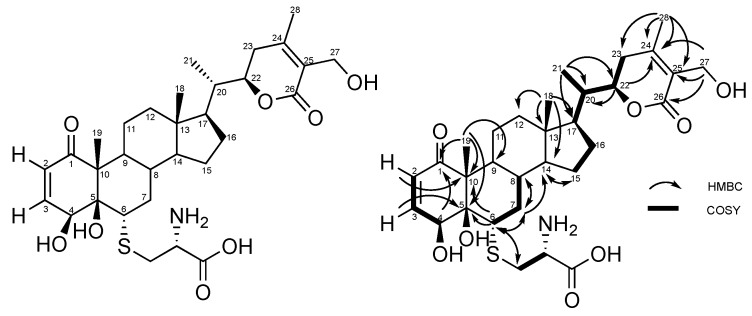
HMBC and COSY correlations of compound **1**.

The ^13^C NMR revealed the presence of 31 carbons, attributed by ^1^H-^13^C EDT-HSQC to: four methyl carbons (δC 10.5, 12.4, 13.6 and 20.3 ppm); seven methylene groups including one sulfur-bond and one oxygen-bond methylene (δC 24.3, 25.2, 28.5, 30.8, 35.3, 38.7 and 56.3 ppm); two methylidene groups (δC 127.5 and 148.8 ppm); nine methine groups including one sulfur-bond, one nitrogen-bond, and two oxygen-bond methines at δC 52.4 (C-6), 55.7, 67.3 (C-4) and 80.1 (C-22) ppm, respectively; and six non-protonated carbons including the cyclohex-2-en-1-one carbonyl at δC 204.1 (C-1), the α,β-unsaturated δ-lactone carbonyl at δC 168.5 (C-26), the cysteine acid function at δC 172.8, and the double bonds of the α, β-unsaturated δ-lactone moiety at δC 126.4 (C-25) and δC 157.8 (C-2). The nJ 1H-13C connectivities provided by the HMBC NMR experiment are listed in Table 1 and depicted in Figure 5.

NMR data recorded for **1** closely resemble those of withaferin A, previously isolated from *Withania somnifera* extract and characterized. Based on the common withanolide scaffold, the 1-conjugated ketone, the 20-unsubstituted methine group, and the 27-hydroxy α, β-unsaturated δ-lactone side chain were similar. Moreover, as in withaferin A, position 4 (δC 67.3 ppm) is hydroxylated. The main differences concern C-5 and C-6 of the epoxide ring in withaferin A. In **1**, C-5 is hydroxylated (δC 81.4 ppm) while C-6 is engaged in a C-S bond of a cysteine moiety, according to the HMBC correlation between the methylidene function of cysteine and C-6.

The cysteine moiety was built according to COSY correlations between methine 3.70 (1H; m) and methylene 2.95 (1H; dd; 14.3, 6.6) and 3.13 (1H; dd; 14.3, 4.7) and the HMBC correlation between methine 3.70 (1H; m) and the carbonyl function at δC 172.8 ppm. Spectroscopic data are provided in the supplementary materials S1 to S8.

Compound **2** was isolated as a white powder. Its molecular formula, C_38_H_55_N_3_O_12_S, was deduced by HRESIMS with *m*/*z* 778.3584 [M + H]^+^. This formula was corroborated by the ^13^C NMR spectrum and revealed 13 degrees of unsaturation. The IR spectrum exhibited characteristic absorption bands at 3300 (OH) as well as 1670 and 1685 cm^−1^ (α,β-unsaturated δ-lactone and carbonyl groups, respectively). These degrees of unsaturation were attributed to the α,β-unsaturated δ-lactone moiety, which is a five-membered ring linked to three six-membered rings, one of them consisting in a cyclohex-2-en-1-one, two carboxylic acids, and one amide group.

**Figure 6:**
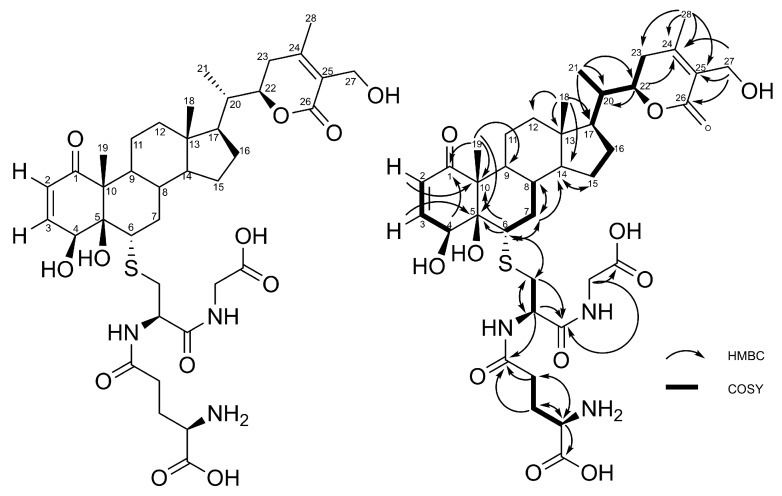
HMBC and COSY correlations of compound **2**.

The ^13^C NMR revealed the presence of 38 carbons, attributed by ^1^H-^13^C EDT-HSQC to: four methyl carbons (δC 10.3, 12.3, 13.6, and 20.2 ppm); 10 methylene groups including one sulfur-bond, one nitrogen-bond, and one oxygen-bond methylene (δC 24.2, 25.2, 27.9, 28.5, 30.8, 33.2, 36.3, 40.3 42.9 and 56.5 ppm); two methylidene groups (δC 127.5 and 148.6 ppm); 10 methine groups including one sulfur-bond, two nitrogen-bond, and two oxygen-bond methines at δC 52.4 (C-6), 55.0, 55.5, 67.3 (C-4), and 80.1 (C-22) ppm, respectively; and nine non-protonated carbons including the cyclohex-2-en-1-one carbonyl at δC 204.1 (C-1), the α,β-unsaturated δ-lactone carbonyl at δC 168.5 (C-26), the glutation acid functions at δC 173.9 and 173.9 ppm, the glutathione amide functions δC 172.9, 172.9, and 175.2 ppm, and the double bonds of the α, β-unsaturated δ-lactone moiety at δC 126.4 (C-25) and δC 157.8 (C-2). nJ 1H-13C connectivities provided by HMBC NMR experiment are listed in Table 2 and depicted in Figure 6.

NMR data recorded for **2** closely resemble those of compound **1**. The main difference between **2** and **1** was the peptide linked to the withanolide core through C-6. According to NRM data (see NMR tables), we linked a glutathione tripeptide moiety to C-6 thanks to the HMBC correlation between the methylidene function of cysteine residue 3.08 (1H; dd; 13.7, 5.7) and 2.81 (1H; dd; 13.7, 7.6) and C-6. Spectroscopic data are provided in the supplementary materials S9 to S16.

### 2.2. Stereochemistry of C-5 and C-6 in CR-591 (***1***) and CR-777 (***2***)

**Figure 7:**
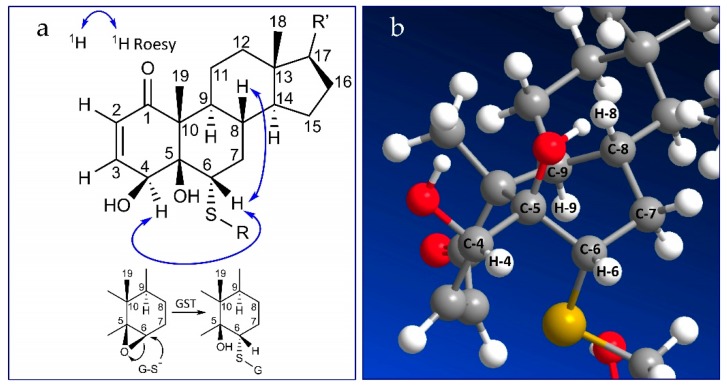
Stereochemistry at C-5 and C6 according to ROE correlations (**a**) and Chem3D structure (**b**).

The ROESY spectrum of compounds **1** and **2** supported by the 3D structures obtained from Chem3D software (ChemBio3D, Perkin Elmer, Waltham, MA, US) allowed us to determine the configuration at C-5 and C6. ROE correlations were observed for both compounds between H-6 and H-4 and between H-6 and H-8. H-9 did not correlate with H-4, H-6, and H-8. According to these data, only the conformation shown in Figure 7 accounts for the observed ROE correlations, corresponding to (*4S*, *5R*, *6S*) stereochemistry.

### 2.3. Mechanism of Formation of CR-591 (***1***) and CR-777 (***2***)

**Figure 8:**
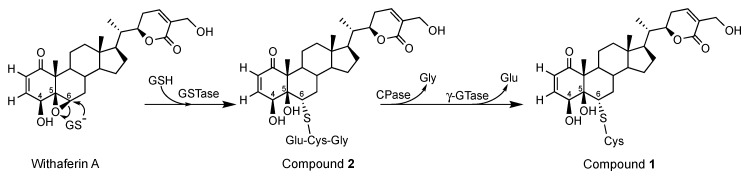
Hypothetic biosynthetic pathway of compounds **1** and **2**.

Among the detoxification processes, the glutathione S-transferases (GSTs) play a central role by catalyzing the conjugation with the ubiquitous tripeptide glutathione (Glu-Cys-Gly), thereby increasing the hydrophilicity of potentially toxic compounds during detoxification. As withaferin A was previously reported for antifungal properties [30,31,32,33,34,35,36], the formation of compounds **1** and **2** through glutathione type conjugates may be due to a detoxification process by *Beauveria bassiana* to escape this antifungal activity. Indeed, glutathione transferase activity was previously reported in *Beauveria bassiana* exposed to toxic compounds [37] which is a common feature in fungi [38]. Figure 8 shows the hypothetic detoxification of withaferin A by *Beauveria bassiana* catalyzed by a glutathione-S-transferase (GST) in which the glutathione (GSH) is attached to carbon C-6 leading to compound **2**. Compound **1** could then form by a successive hydrolysis of the glycine by a carboxypeptidase (CPase) and glutamate by a γ-glutamyl-transferase (γ-GTase). Similar pathways were largely reported during the detoxification of different families of pesticides by phytopathogens, and the involved enzymes cloned and identified [38,39].

We have previously reported a neutraceutical preparation produced by the bioconversion with *B. bassiana* of three Ayurvedic plants *Withania somnifera*, *Bacopa monnieri,* and *Emblica officinalis*. This preparation was named SNC1 and its impact on angiogenesis and neuronal disorders was previously investigated [26,27,28,29]. Based on our preliminary results on the neuroprotective effects of SNC1, as well as on the preliminary investigation of the compounds **1** and **2**, the potential neuroprotective effect of CR-777, the glutathione conjugate of withaferin A, was evaluated.

### 2.4. Impact on Parkinson’s Mimetics

The neurotoxic compound MPTP (1-methyl-4-phenyl-1,2,3,6-tetrahydropyridine) is converted to MPP+ by the astrocytic monoamine oxidase-B. MPTP is one of the most studied neurotoxic compounds as it induces parkinsonian syndrome- and neurodegeneration-like effects in dopaminergic neurons (Visanji et al., 2008) [40]. These effects are accompanied by most phenotypic disorders that characterize PD, including tremor, rigidity, slowness, and postural instability. Specifically, MPP+ accumulates in the mitochondria and inhibits complex I of the respiratory chain with an increase in superoxide formation. Furthermore, MPP+ uptake into dopaminergic neurons triggers vesicular dopamine release. In the presence of iron and hydrogen peroxide, dopamine is oxidized to 6-hydroxydopamine (6-OHDA) which cyclizes to give aminochrome which then directly inhibits respiratory chain complex I [41].

α-Synuclein is a small (140-amino-acid-long) protein located in the presynaptic terminals of neurons and involved in the plasticity of the synapse and vesicular traffic during neurotransmitter release. In PD, around 90% of cellular α-synuclein is phosphorylated (as compared to ~4% in healthy people) resulting in the accumulation of α-synuclein inclusions and neurotoxicity [42]. Addition of exogenous α-Syn to neurons in culture is suspected to be toxic by mimicking self-association to aggregates, which then have a direct impact on the mitochondrial production of reactive oxygen species (ROS).

The cytotoxicity analysis of CR-777 (**2**) and withaferin A using primary HUVECs shows that CR-777 is less toxic, with an IC_50_ of 27.1 μM ± 5.4 compared to 2.1 μM ± 0.02 for withaferin A.

**Figure 9:**
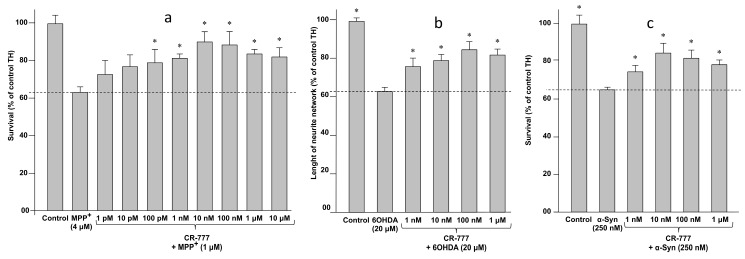
Cytoprotective effect of CR-777 (**2**) on dopaminergic neurons exposed 1 h before cell exposure to (**a**) 1-methyl-4-phenylpyridinium (MPP+), (**b**) 6-hydroxydopamine (6-OHDA) or (**c**) α-synuclein (α-Syn). Data are expressed as percentage of control as mean ± SEM (100% = no MPP+, no compound). Statistical analyses were performed using the Graph pad prism for one-way ANOVA followed by Dunnett’s test. * *p* < 0.05 was considered significant.

When mesencephalic neurons were treated for various periods with different concentrations of each of the three aforementioned neuro-toxicants, i.e., MPP+, 6-OHDA, and α-Syn, they underwent cell death at a rate of ~30–40% (MPP+ 4 µM/48 h, 6-OHDA 20 µM/48 h, α-synuclein 250 nM/24 h). Notably, compound **2**, added 1 h before cell exposure to neurotoxic MPP+, α-Syn or 6-OHD, showed significant neuro-protection, with a maximal effect observed at the dose of 10 nM (Figure 9). At these concentrations, withaferin A did not exhibit any neuroprotective effect, while showing significant toxicity at 1 μM and a complete cell death at 10 μM.

**Figure 10:**
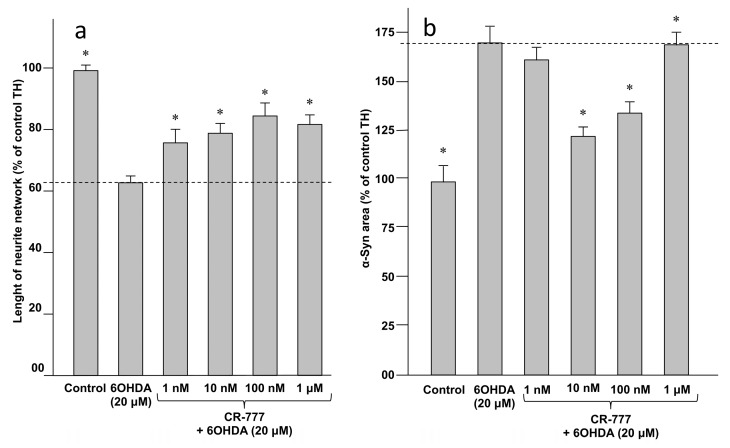
CR-777 (**2**) (**a**) protects neurite network and (**b**) suppress α-Syn upregulation after 6-OHDA injury. Results are given as mean ± SEM (vs. control set to 100%). * *p* < 0.05 vs. compound **2** (one-way ANOVA followed by PLSD Fisher’s test).

**Figure 11:**
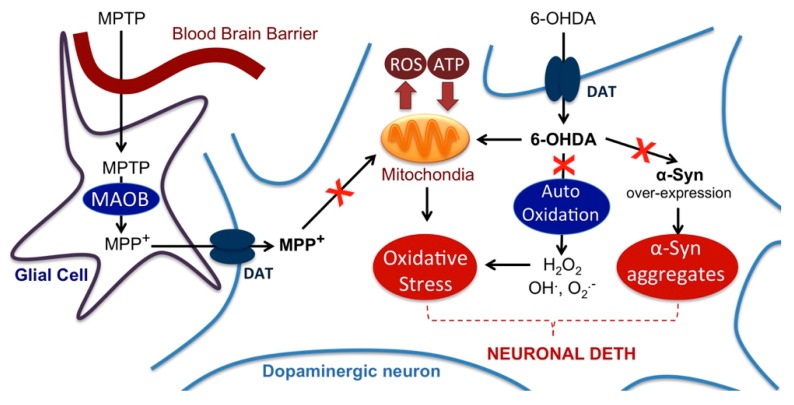
Model illustrating the action of the different neurotoxins used to mimic Parkinson’s disease (PD) injury on neuronal cells. The red cross indicates possible pathways that are inhibited by CR-777 according to the results reported above. DAT: dopamine transporter; MAOB: monoamine oxidase B; ROS: reactive oxygen species.

Retraction of neurite network and overexpression of synuclein (synucleinopathy) are two known pathological features of PD, being induced by several types of injuries including 6-OHDA treatment (Figure 9). Co-exposure of neuron cells to both 6-OHDA and compound **2** successfully protected the integrity of the neurite network of dopaminergic neurons at a 1-nM concentration, and at the optimal concentration of 10 nM suppressed 6-OHDA-mediated α-Syn upregulation (Figure 10). Taken together these findings indicate that CR-777 (**2**) is neuroprotective against mitochondrial injury on dopaminergic neurons (Figure 11).

In order to investigate whether classical neuroprotective pathways (i.e., RAS, PI3K/AKT, BCL2 and PPAR-γ) [43,44] are involved in the activities of compound **2**, primary cultures of rat embryonic cortical neurons were exposed to 6-OHDA and compound **2**, in the presence (or not) of specific inhibitors of neuroprotective pathways (Table 3).

**Figure 12:**
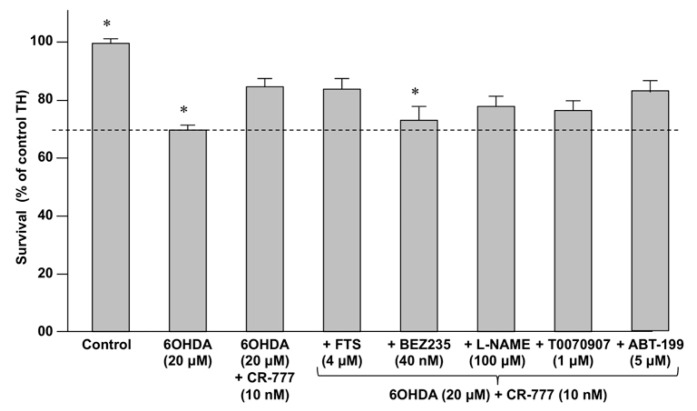
Impact of compound **2** on neurons survival in the presence of specific inhibitors (see Table 3) following co-exposure with 6-OHDA (20 µM, 24 h). Results are given as mean ± SEM (vs. control set to 100%). * *p* < 0.05 vs. compound **2** (one-way ANOVA followed by PLSD Fisher’s test).

As shown in Figure 12, the inhibition of the PI3K/mTOR pathway by BEZ-235 abolished the neuroprotective effect of compound **2** against 6-OHDA. This finding indicates that the CR-777-induced neuroprotection likely involves, at least in part, the activation of the PI3K/mTOR pathway which plays a key role in the development and proper functioning of the brain [43].

**Figure 13:**
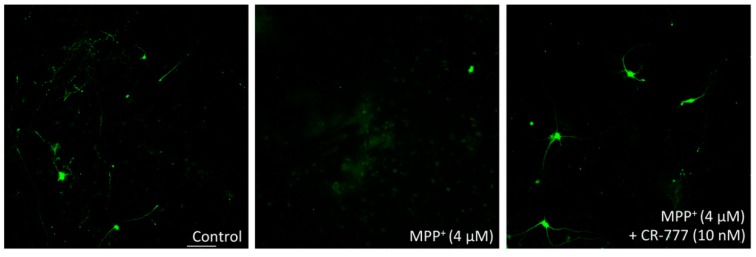
Immunofluorescence images of neurons stained for microtubule-associated protein 2 (MAP2; scale bar 100 µm).

The reduction in microtubule-associated protein 2 (MAP2) is considered as a marker of injured cortical neurons, engaged irreversibly in cell death. In parallel, reduced expression of tyrosine hydroxylase (TH), which catalyzes the conversion of L-tyrosine to l-3,4-dihydroxyphenylalanine (L-DOPA), is a marker of dopaminergic neuron injury. Figure 13 represents immunofluorescence images of neurons stained for MAP2 expression. As is evident, CR-777 at the concentration of 10 nM protects the neurons from injury by MPP+.

## 3. Discussion

*Withania somnifera* (Ashwagandha) is an Ayurvedic medicinal plant with pleiotropic properties, including neuro-protective action [6,7]. The plant contains a large diversity of withanolides and withanosides expected to be responsible for the neuroprotective activity. *Withania somnifera*, together with *Bacopa monnieri* and *Emblica officinalis,* was bioconverted by *Beauveria bassiana* to lead to a nutraceutical preparation called SNC1. The impact of this preparation on angiogenesis and neuronal disorders were investigated and SNC1 was recently launched on the market as Ethnodyne Visio^®^ for age-related macular degeneration and Ethnodyne-Neuro^®^ for neurodegerative diseases.

In order to identify the bioactive compounds in the bioconversion mixture, the three plant extracts were incubated separately with *B. Bassiana.* Analytical investigations indicate significant changes in the chromatograms for the *W. somnifera* extract. Two new compounds were isolated from the biotransformed extracts of *W. somnifera* and fully characterized as the cysteine and glutathione conjugates of withaferin A. Thus, withaferin A was purified from an enriched extract of *W. somnifera* and submitted to the *W. somnifera* to access significant quantities of the target compounds. Cysteine and glutathione conjugates of withaferin A were named CR-591 and CR-777, respectively. According to preliminary results, the glutathione conjugate CR-777 exhibited significant neuro-protective properties from damage caused by different toxins mimicking molecular symptoms of AD and PD. Specifically, mesencephalic neuron injury causing around 30–40% cell death using MPP^+^, 6-OHDA, and α-Syn, was reversed with a nanomolar concentration of CR-777 added 1 h before injury. Furthermore CR-777 protects the integrity of the neurite network and reduced the α-Syn overexpression induced by 6-OHDA. By using different inhibitors of cell signaling pathways, these neuro-protective properties seem to suppress oxidative stress and α-Syn aggregation via the induction of the cytoprotective PI3K/mTOR pathway.

Similarly, the use of glutamate and Aβ mimics of AD induced around 30% cell death, which was reversed by pretreatment with nanomolar concentrations of CR-777. Both molecules also induced a ~40% reduction of the neurite network, which was largely attenuated by pretreatment with CR-777. Interestingly, the usage of signaling pathway inhibitors indicates the involvement of two cell survival pathways, namely PI3K/mTOR and the ERK/RAS, as key mediators of the CR-777 activity. Staining experiments indicate a combined beneficial effect of CR-777 involving the inhibition of TAU Protein phosphorylation and a decrease in the expression of caspase-3 expression.

The involvement of mammalian GST in the detoxification of xenobiotic compounds is well documented. Thus, glutathione conjugation with withaferin A to CR-777 may also take place in human body after oral administration of *W. somnifera* preparations to PD and AD patients.

Our running efforts are focused on the large-scale production of CR-777 by direct and selective conjugation of glutathione with withaferin A. This will allow advanced cell-based and molecular investigations as well as clinical trials with PD and AD patients.

## 4. Materials and Methods

### 4.1. Analytical and Structure Elucidation Methods of Target Molecules

Optical rotations [α]_D_ were measured using an Anton Paar MCP-300 polarimeter at 589 nm. The IR spectra were obtained using a Perkin-Elmer Spectrum 100 model instrument. NMR experiments were performed using a Bruker Avance 500 MHz spectrometer (Bruker Biospin, Wissembourg, France). All the spectra were acquired in CD_3_OD (δ_H_ 3.31 ppm and δ_C_ 49.15 ppm) at room temperature. High-resolution mass spectra were obtained on a Waters LCT Premier XE spectrometer with electrospray-time of flight (ESI-TOF) by direct infusion of the purified compounds. Pre-packed silica gel Redisep columns were used for flash chromatography using a Combiflash-Companion (Serlabo, Entraigues sur La Sorgue, France). All other chemicals and solvents were purchased from SDS (Peypin, France).

The analytical HPLC system consisted of an Alliance Waters 2695 controller coupled with a photo diode array (PDA, Waters 2996), an evaporative light-scattering detector (ELSD, Waters 2424), and a mass detector (Waters, QDa). A Sunfire analytical C18 column (4.6 × 150 mm, 3.5 μm) was used with a flow rate of 0.7 mL/min. The elution gradient consisted of 100% water (+0.1% formic acid) to 100% acetonitrile (+0.1% formic acid) in 40 min, then 10 min at 100% acetonitrile (+0.1% formic acid). Preparative HPLC was performed on a Sunfire C18 column (10 × 250 mm, 5 μm) using the Waters autosampler 717, pump 600, photodiode array detector 2996, and an ELSD detector 2420 (Waters, Guyancourt, France). The 4 mL/min flow rate gradient was from 20 to 24% of acetonitrile with 0.1% formic acid in 20 min, followed by an isocratic at 24% of acetonitrile with 0.1% formic acid for 10 min.

### 4.2. Plant Extraction Procedure

Dry roots of *Withania somnifera* were powdered mechanically, and extracted by high-pressure static extraction using the in-house Zippertex technology [26]. Then, 650 g were extracted twice with 4 L of a hydro-alcoholic mixture (ethanol/water 60:40) at 40 °C under static pressure in the 10-L cell of the Zippertex (100 bars, 30 min). Ethanol was evaporated under reduced pressure and the remaining 1.4 L aqueous fraction (representing 86 g of dry extract) was submitted to solid-phase extraction (SPE) using 150 g of Amberlite XAD-16 resin (Dow, Saint-Denis, France). The resin/extract mixture was gently stirred for 2 h, and then the water was removed by filtration and the resin washed twice with 1 L of distilled water. The resin was recovered in 500 mL of MeOH, filtered and the methanol evaporated under reduced pressure to give 8 g (9% yield from the hydro-alcoholic extract) of the withanoside/withanolide-enriched extract named WE-SPE.

### 4.3. Cultivation of B. bassiana

Fungal strain *Beauveria bassiana* ATCC 7159 (LGC Standards, Molsheim, France) was cultivated in a liquid medium consisting of (per liter of water) 10 g of corn steep liquor (Roquette, Lestrem, France), 0.5 g of KH_2_PO_4_, 1 g of K_2_HPO_4_, 1 g of MgSO_4_, 2 g of NaNO_3_, 0.5 g of KCl, 0.02 g of FeSO_4_, and 30 g of glucose. The culture was incubated at 27 °C under shaking at 150 rpm. After 3 days, the biomass was recovered by filtration. The wet biomass was used fresh for the resting-cell bioconversion experiment (65 g/L).

### 4.4. Fermentation of the Plant Extract

Here, 20 g of WE-SPE extract of *W. somnifera* were mixed with 120 g of wet *B. bassiana* in a 4-L Erlenmayer flask containing 2 L of water and 100 g of glucose. The suspension was incubated at 27 °C and 150 rpm. After 5 days, the mixture was filtered through 0.22-µm membrane filters (AIT, Corbeil-Essonnes, France) and lyophilized while waiting for the fractionation and purification steps.

### 4.5. Fractionation of Extract and Purification of Target Compounds

Here, 4.8 g of the fermented extract were fractionated by flash chromatography on a CombiFlash Companion (Entraigues-sur-la-Sorgue, France) using a pre-filled silica column (GraceResolv 80 g) with a flow rate of 40 mL/min. The solvent used consisted on a dichloromethane (DCM)/MeOH mixture. The gradient used was: 0–10 min 90/10, 10–20 min 85/15, 20–75 min 80/20. Collection tubes were pooled in seven fractions. Further purifications were made by preparative HPLC as reported above.

CR-591 (1)-enriched fractions (360 mg) were dissolved in a water/methanol mixture (at 100 mg/mL) and purified by semi-preparative HPLC (operating conditions previously mentioned), offering 15 mg of pure CR-591.

CR-777 (2)-enriched fractions (420 mg) were dissolved in a water/methanol mixture (at 100 mg/mL) and purified by semi-preparative HPLC (operating conditions previously mentioned) offering 26 mg of pure CR-777.

### 4.6. Isolation of *Withaferin A*

Dry roots of *Withania somnifera* were extracted as previously described. The ethanol was evaporated under reduced pressure and the remaining aqueous fraction extracted with dichloromethane. The organic layer was dried on sodium sulfate and evaporated to give a light-brown solid consisting mainly of withanolides. Three grams of this extract were fractionated by flash chromatography on a CombiFlash Companion (Serlabo Techbologies, Entraigues-sur-la-Sorgue, France) using a pre-filled silica column (GraceResolv 80 g) with a flow rate of 40 mL/min. The solvent used consisted of a heptane/ethyl acetate mixture. The gradient used was: 0–2 min 100/0, 2–15 min 60/40, 15–30 min 40/60, 30–90 min 20/80.

The withaferin A-enriched fraction (350 mg) was purified by semi-preparative HPLC on a reverse-phase column Sunfire III C18 (10 × 250 mm, 5 μm, Waters, Guyancourt, France), using an isocratic method with 38% acetonitrile. UV Detection was performed at 230 nm. After concentration under reduced pressure, 180 mg of pure Withaferin A were obtained.

### 4.7. Compliance with the Rules for Laboratory Animal Use

The collection of embryos was carried out in accordance with the National Institutes of Health Guide for the Care and Use of Laboratory Animals and followed current European Union regulations (Directive 2010/63/EU). The process was supervised and approved by the local head of the veterinary services of the Bouches-du-Rhône (agreement number A1301310).

### 4.8. Culture of Mesencephalic Neurons for MPP+ Injury

Rat dopaminergic neurons were cultured as previously described [45,46]. Briefly, the midbrains obtained from 15-day old rat embryos (Janvier Labs, Le Genest-Saint-Isle, France) were dissected under a microscope. The embryonic midbrains were removed and placed in ice-cold medium of Leibovitz (L15, Thermo-fisher, Les Ulis, France) containing 2% of penicillin–streptomycin (PS, Dutsher, Brumath, France) and 1% bovine serum albumin (BSA, Dutsher,). The ventral portion of the mesencephalic flexure, a region of the developing brain rich in dopaminergic neurons, was used for the cell preparations.

The midbrains were dissociated by trypsinization for 20 min at 37 °C (Trypsin 0.05% EDTA 0.02%, Dutsher). The reaction was stopped by the addition of Dulbecco’s modified Eagle’s medium (DMEM, Thermo-fisher) containing DNAase I grade II (0.1 mg/mL, Thermo-fisher) and 10% fetal calf serum (FCS, Thermo-fisher). Cells were then mechanically dissociated by three passages through a 10-mL pipette. Cells were then centrifuged at 180× *g* for 10 min at +4 °C on a layer of BSA (3.5%) in L15 medium. The supernatant was discarded and the cell pellets were re-suspended in a defined culture medium consisting of Neurobasal (Invitrogen, Paris, France) supplemented with B27 (2%, Invitrogen), L-glutamine (2 mM, Thermo-fisher, Les Ulis, France), 2% penicillin–streptomycin solution (PS), 10 ng/mL brain-derived neurotrophic factor (BDNF, Dutsher), and 1 ng/mL of glial-derived neurotrophic factor (GDNF, Thermo-fisher). Viable cells were counted in a Neubauer cytometer using the trypan blue exclusion test. The cells were seeded at a density of 40,000 cells/well in 96 well plates pre-coated with poly-L-lysine (Corning Biocoat, Boulogne-Billancourt, France) and maintained in a humidified incubator at 37 °C in 5% CO_2_/95% air atmosphere. Half of the medium was changed every 2 days with fresh medium. Six wells per condition were assessed.

### 4.9. Culture of Mesencephalic Neurons for α-Synuclein Injury

Rat dopaminergic neurons were cultured as previously described [47]. Briefly, the midbrains obtained from 14-day-old rat embryos (Janvier Labs) were dissected under a microscope. The embryonic midbrains were removed and placed in ice-cold medium of Leibovitz containing 2% penicillin–streptomycin and 1% bovine serum albumin (BSA). The ventral portion of the mesencephalic flexure, a region of the developing brain rich in dopaminergic neurons, was used for the cell preparations.

The midbrains were dissociated by trypsinization for 20 min at 37 °C (Trypsin 0.05% EDTA 0.02%, Dutsher, Brumath, France). The reaction was stopped by the addition of Dulbecco’s modified Eagle’s medium (DMEM, Dutsher) containing DNAase I grade II (0.1 mg/mL, Dutsher) and 10% fetal calf serum (FCS, Thermo-fisher). Cells were then mechanically dissociated by three passages through a 10-mL pipette. Cells were then centrifuged at 180× *g* for 10 min at +4 °C on a layer of BSA (3.5%) in L15 medium. The supernatant was discarded and the cell pellets were re-suspended in a defined culture medium consisting of Minimum essential medium (MEM, Dutsher) containing 10% heat-inactivated FCS, 10% heat-inactivated horse serum (HS, Thermo-fisher), 1 g/L glucose (Sigma Aldrich), 1 mM sodium pyruvate (Sigma Aldrich, Saint-Quentin-Fallavier, France), 100 µM non-essential amino acids (Dutsher, Brumath, France), L-glutamine (2 mM, and 2% PS solution. Viable cells were counted in a Neubauer cytometer using the trypan blue exclusion test. The cells were seeded at a density of 80,000 cells/well in 96 well-plates pre-coated with poly-L-lysine and maintained in a humidified incubator at 37 °C in 5% CO_2_/95% air atmosphere. Half of the medium was changed every 2 days with fresh medium. Six wells per condition were assessed.

### 4.10. CR-777 Exposure

CR-777 (**2**) was dissolved in culture medium and pre incubated for 1 h before toxin application at different concentrations.

- MPP+ injury: On day 6 of culture, the medium was removed and fresh medium was added, without or with MPP+ (Sigma Aldrich), at 4 µM diluted in control medium with or without CR-777 for 48 h.
- 6-OHDA injury: On day 6 of culture, the medium was removed and fresh medium was added, without or with 6-OHDA (Sigma Aldrich), at 20 µM diluted in control medium with or without CR-777 for 48 h.
- α-Synuclein injury: On day 7 of culture, the medium was removed and fresh medium was added, without or with alpha synuclein (rPeptide), at 250 nM diluted in control medium with or without CR-777 for 24 h.

### 4.11. Immunostaining: TH Positive Neron (Dopaminergic Neurons)

After treatment, cells were fixed by a solution of 4% paraformaldehyde (PFA, Sigma) in PBS (PAN), pH = 7.3 for 20 min at room temperature. The cells were washed again twice in PBS, and then were permeabilized and non-specific sites were blocked with a solution of PBS containing 0.1% of saponin (Sigma) and 1% FCS for 15 min at room temperature. Then, cells were incubated with monoclonal anti-tyrosine hydroxylase (TH, Sigma) antibody produced in mouse at dilution of 1/10,000 in PBS containing 1% FCS, and 0.1% saponin for 2 h at room temperature. This antibody was revealed with Alexa Fluor 488 goat anti-mouse IgG (Molecular Probe) at a dilution of 1/800 in PBS containing 1% FCS and 0.1% saponin for 1 h at room temperature.

Tyrosine hydroxylase is involved in the conversion of phenylalanine to dopamine. As the rate-limiting enzyme in the synthesis of catecholamines, tyrosine hydroxylase has a key role in the physiology of adrenergic neurons. Tyrosine hydroxylase is regularly used as a marker for dopaminergic neurons, which is particularly relevant for research on Parkinson’s disease.

### 4.12. Immunostaining: Overepression of α-Syuclein (Dopaminergic Neurons)

After treatment, cells were fixed by a solution of 4% paraformaldehyde (PFA, Sigma) in PBS (PAN), pH = 7.3, for 20 min at room temperature. The cells were washed again twice in PBS, and then were permeabilized and non-specific sites were blocked with a solution of PBS containing 0.1% saponin (Sigma) and 1% FCS for 15 min at room temperature. Then, cells were incubated with:

- Monoclonal anti-tyrosine hydroxylase (TH, Sigma) antibody produced in mice at a dilution of 1/10,000 in PBS containing 1% FCS and 0.1% saponin for 2 h at room temperature. This antibody was revealed with Alexa Fluor 488 goat anti-mouse IgG (Molecular Probe) at the dilution of 1/800 in PBS containing 1% FCS and 0.1% saponin for 1 h at room temperature.
- Monoclonal anti-alpha-synuclein (Cell Signaling) antibody produced in rabbit at a dilution of 1/200 in PBS containing 1% FCS and 0.1% saponin for 2 h at room temperature. This antibody was revealed with Alexa Fluor 568 goat anti-rabbit IgG (Molecular Probe) at the dilution of 1/800 in PBS containing 1% FCS and 0.1% saponin for 1 h at room temperature.
- Immunostaining: MAP-2 tau phosphor Ser214, Thr212 (cortical neurons)

After intoxication, the cell culture supernatant was taken out and the cortical neurons were fixed by a cold solution of ethanol (95%) and acetic acid (5%) for 5 min at −20 °C. After permeabilization with 0.1% of saponin, cells were incubated for 2 h with:

- Mouse monoclonal antibody anti phosphoT (phospho Thr212/Ser214; AT100) at a dilution of 1/400 in PBS containing 1% fetal calf serum and 0.1% saponin.
- Chicken polyclonal antibody anti microtubule-associated-protein 2 (MAP-2) at a dilution of 1/1000 in PBS containing 1% fetal calf serum and 0.1% saponin (this antibody stains specifically cell bodies and neurites, allowing study of neuronal cell survival and neurite network).

These antibodies were revealed with Alexa Fluor 488 goat anti-mouse IgG and Alexa Fluor 568 goat anti-chicken IgG at the dilution 1/400 in PBS containing 1% FCS and 0.1% saponin for 1 h at room temperature.

### 4.13. Analysis

The immunolabeled cultures were automatically examined with ImageXpress (Molecular Devices) equipped with a LED at ×10 (for dopaminergic neuron) and ×20 (for cortical neuron) magnification. For each condition (six culture wells), 20 automatic fields per well (for dopaminergic neurons) and 30 automatic fields per well (for cortical neurons) (representing ~80% of the total surface of the well) were analyzed.

The total number of TH neurons, the MAP-2 positive neurons, the neurite network, the alpha-syn into dopaminergic neurons, and hyperphophorylation of T protein (pT) into neurons (pT/MAP-2) were automatically analyzed using MetaXpress software (Molecular Devices).

### 4.14. Statistical Analysis

Data were expressed in percentage of control conditions (no intoxication, no toxin = 100%) in order to express the toxin injury. All values were expressed as mean +/− SEM (Standard Error mean) of the six wells. Graphs and statistical analyses were performed in the different conditions (ANOVA followed by Fisher’s test when allowed).

## Figures and Tables

**Table 1:** NMR spectroscopic data of compound **1**.

Position	δC	δH (mult; J in Hz)	HMBC
1	204.1	-	
2	127.5	5.89 (1H; dd; 10.2; 2.0)	C-4, C-10
3	148.8	6.58 (1H; dd; 10.2, 1.9)	C-1, C-5
4	67.3	4.89 (1H; m)	C-1, C-2, C-3, C-6
5	81.4	-	
6	52.4	3.02 (1H; d; 12.3)	C-4, C-5, C-7, C-10, Cys (35,3)
7	38.7	2.19 (1H; m), 1.55 (1H; m)	C-6, C-8, C-9, C-14
8	36.7	1.67 (1H; m)	
9	47.0	1.39 (1H; m)	
10	59.3	-	
11	28.4	1.81 (1H; m), 1.42 (1H; m)	
12	40.5	1.14 (2H; m)	
13	44.4	-	
14	56.3	1.18 (1H; m)	C-15
15	25.2	1.70 (1H; m), 1.27 (1H; m)	C-14
16	24.3	1.39 (1H; m), 0.90 (1H; m)	C-13
17	53.1	1.23 (1H; m)	
18	12.4	0.75 (3H; s)	C-12, C-13, C14, C-17
19	10.5	1.25 (3H; s)	C-1, C-5, C-9, C-10
20	40.3	1.94 (1H; m)	
21	13.6	0.99 (3H; d; 6.8)	C-17, C-20, C-22
22	80.1	4.44 (1H; d; 13.4)	C-20, C-24
23	30.8	2.52 (1H; d; 17.3), 2.16 (1H; m)	C-22, C-24, C-25
24	157.8	-	
25	126.4	-	
26	168.5	-	
27	56.5	4.34 (2H; m)	C-24, C-25, C-26
28	20.3	2.09 (3H; s)	C-23, C-24, C-25
L-Cys	35.3	3.13 (1H; dd; 14.3, 4.7), 2.95 (1H; dd; 14.3, 6.6)	C-6, Cys (55,7)
	55.7	3.70 (1H; m)	Cys (35,3), Cys (172,8)

**Table 2:** NMR spectroscopic data of compound **2**.

Position	δC	δH (mult; J in Hz)	HMBC
1	204.2	-	
2	127.5	5.89 (1H; dd; 10.2; 2.0)	C-4, C-10
3	148.6	6.57 (1H; dd; 10.2, 1.9)	C-1, C-5
4	67.3	4.90 (1H; m)	C-1, C-2, C-3, C-6
5	81.3	-	
6	52.7	3.16 (1H; d; 12.3)	C-4, C-5, C-7, Cys (36.3)
7	38.5	2.15 (1H; m), 1.51 (1H; m)	C-6, C-8, C-9, C-14
8	36.6	1.66 (1H; m)	
9	47.0	1.39 (1H; m)	
10	59.2	-	
11	28.5	1.81 (1H; m), 1.40 (1H; m)	
12	40.3	1.95 (1H; m)	
13	44.3	-	
14	56.3	1.18 (1H; m)	C-15
15	25.2	1.70 (1H; m), 1.29 (1H; m)	C-14
16	24.2	1.38 (1H; m), 0.89 (1H; m)	C-13
17	53.1	1.23 (1H; m)	
18	12.3	0.75 (3H; s)	C-12, C-13, C14, C-17
19	10.3	1.25 (3H; s)	C-1, C-5, C-9, C-10
20	40.3	1.94 (1H; m)	
21	13.6	0.99 (1H; d; 6.8)	C-17, C-20, C-22
22	80.1	4.44 (1H; d; 13.4)	
23	30.8	2.52 (1H; d; 17.3), 2.14 (1H; m)	C-22, C-24, C-25
24	157.8	-	
25	126.4	-	
26	168.5	-	
27	56.5	4.34 (2H; m)	C-24, C-25, C-26
28	20.2	2.09 (3H; s)	C-23, C-24, C-25
L-Cys	36.3	3.08 (1H; dd; 13.7, 5.7), 2.81 (1H; dd; 13.7, 7.6)	C-6, Cys (55.0), Cys (172.9)
	55.0	4.59 (1H; t; 6.)	Cys (36.3), Cys (172.9), Glu (175.2)
	172.9	-	
L-Glu	27.9	2.13 (2H; m)	Glu (55.5), Glu (175.2)
	33.2	2.54 (2H; m)	Glu (55.5), Glu (175.2)
	55.5	3.67 (1H; m)	Glu (27.9), Glu (33.2), Glu (173.9)
	173.9	-	
	175.2	-	
L-Gly	42.9	3.87 (2H, m)	Cys (172.9), Gly (173.9)
	172.9	-	
	173.9	-	

**Table 3:** Pharmacological agents used to determine the mode of action of compound **2**.

Inhibitor	Activity
Farnesylthiosalicylic acid (FTS)	Inhibits the Ras/Raf pathway by disrupting Ras at the membrane.
BEZ-235	Dual PI3K/mTOR inhibitor
T0070907	Selective PPAR-γ inhibitor
Nitro-L-arginine methyl ester (L-NAME)	Inhibitor of nitric oxide synthase (NOS).
ABT-199	Inhibitor of BLC2

## Supplementary Materials

The following are available online. S1 to S8, Spectroscopic characterization of compound **1**. S9 to S16 Spectroscopic characterization of compound **2**.
