# Supplementary material for: Neuroprotective Effect of CR-777, a Glutathione Derivative of Withaferin A, Obtained through the Bioconversion of Withania somnifera (L.) Dunal Extract by the Fungus Beauveria bassiana
Source: Molecules. 2019 Dec 16;24(24):4599. doi: 10.3390/molecules24244599 (PMC6943490; doi:10.3390/molecules24244599)
Supplement: Supplementary file 1 [file molecules-24-04599-s001.pdf]

Supplementary information

Neuroprotective effect of CR-777, the glutathione derivative of withaferin A, obtained through the bioconversion of *Withania somnifera* (L.) Dunal extract by the fungus *Beauveria bassiana*

Chérif Rabhi,[1] Guillaume Arcile,[2] Géraldine Le Goff,[2] Christian Da Costa Noble,[1] and Jamal Ouazzani [2*]

[1] Laboratoire Ethnodyne, 151 Boulevard Haussmann – 75008, Paris, France. c.rabhi@ethnodyne.com (C.R.); cdcn@horus-finance.com (C.D.C.N.)

[2] Institut de Chimie des Substances Naturelles ICSN, Centre National de la Recherche Scientifique, Avenue de la Terrasse 91198, Gif-sur-Yvette, France. guillaume.arcile@cnrs.fr (G.A.); geraldine.legoff@cnrs.fr (G.L.G.); jamal.ouazzani@cnrs.fr (J.O.).

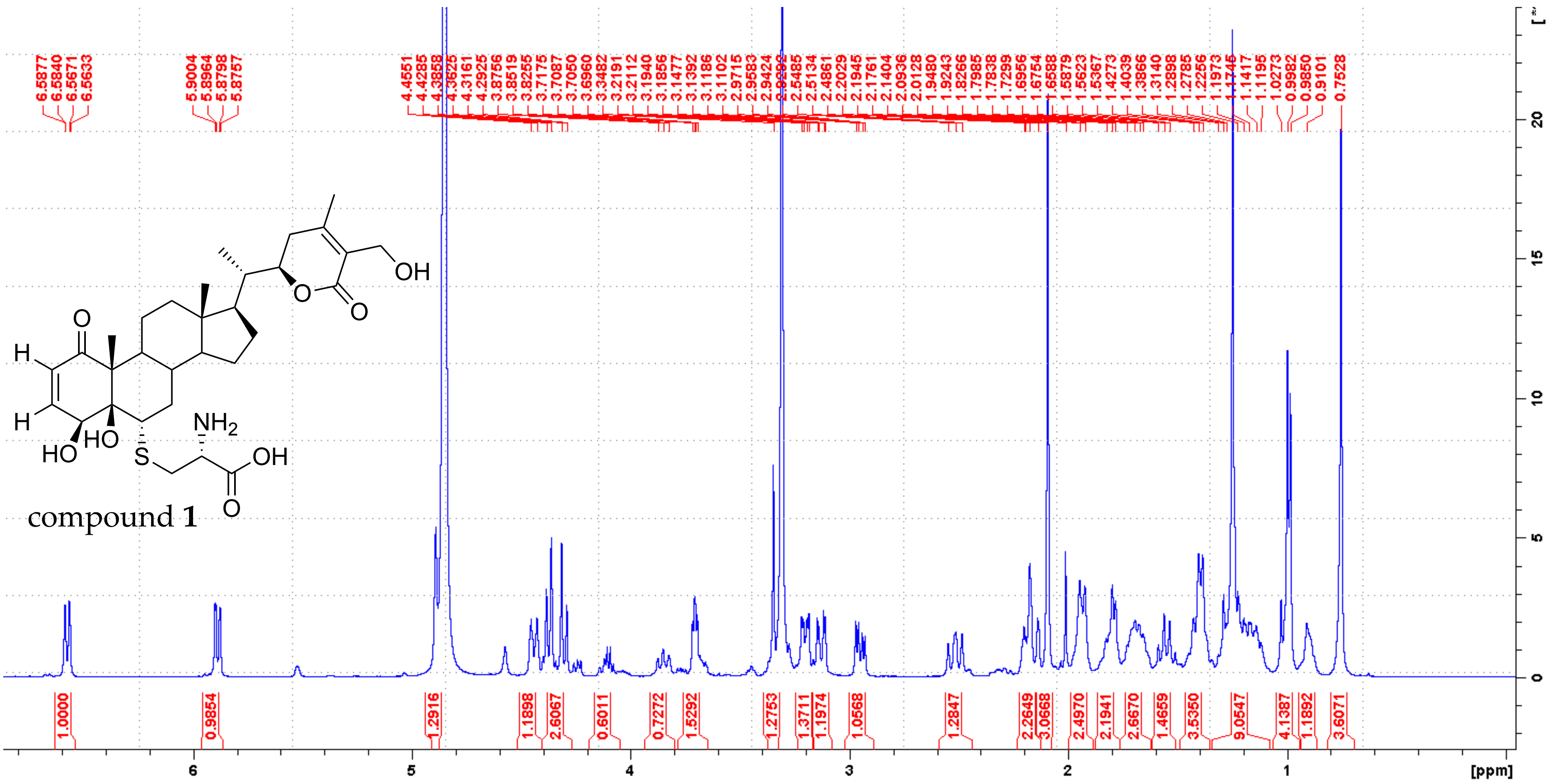

S1. ^{1}H-NMR spectrum (500 MHz, MeOD) of compound **1**

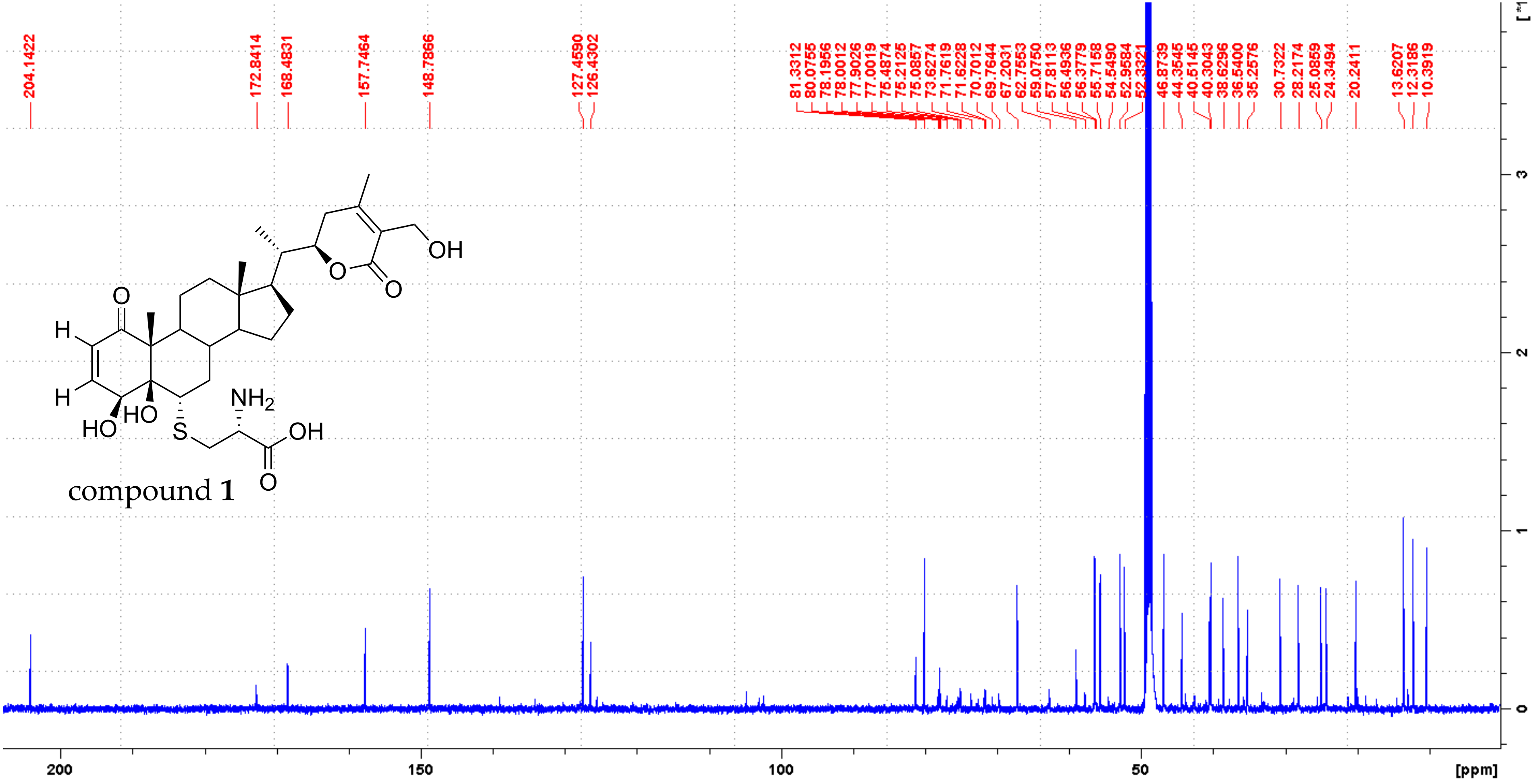

S2. ^{13}C-NMR spectrum (125 MHz, MeOD) of compound **1**

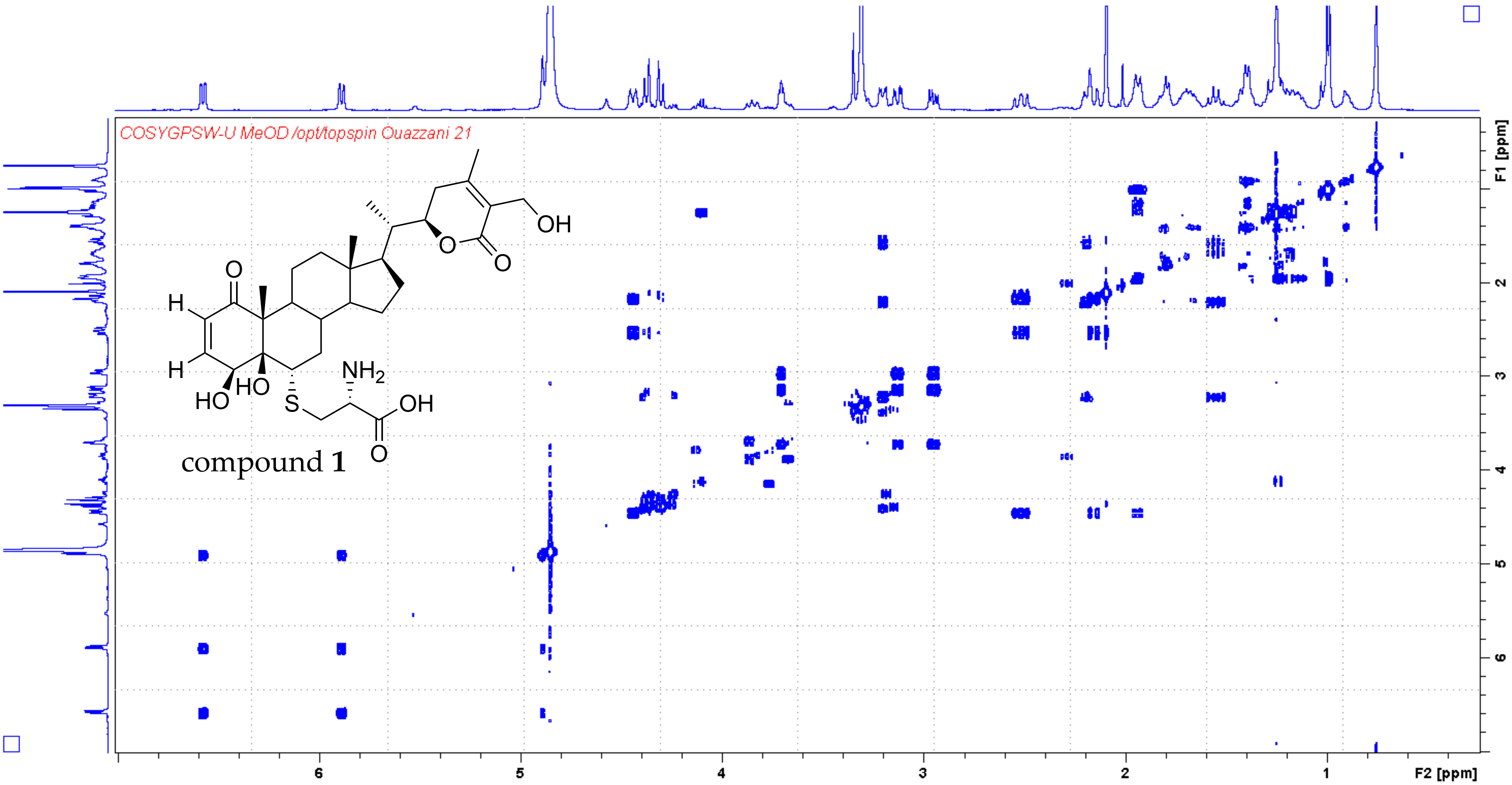

S3. ^1H-^1H COSY NMR spectrum (500 MHz, MeOD) of compound **1**

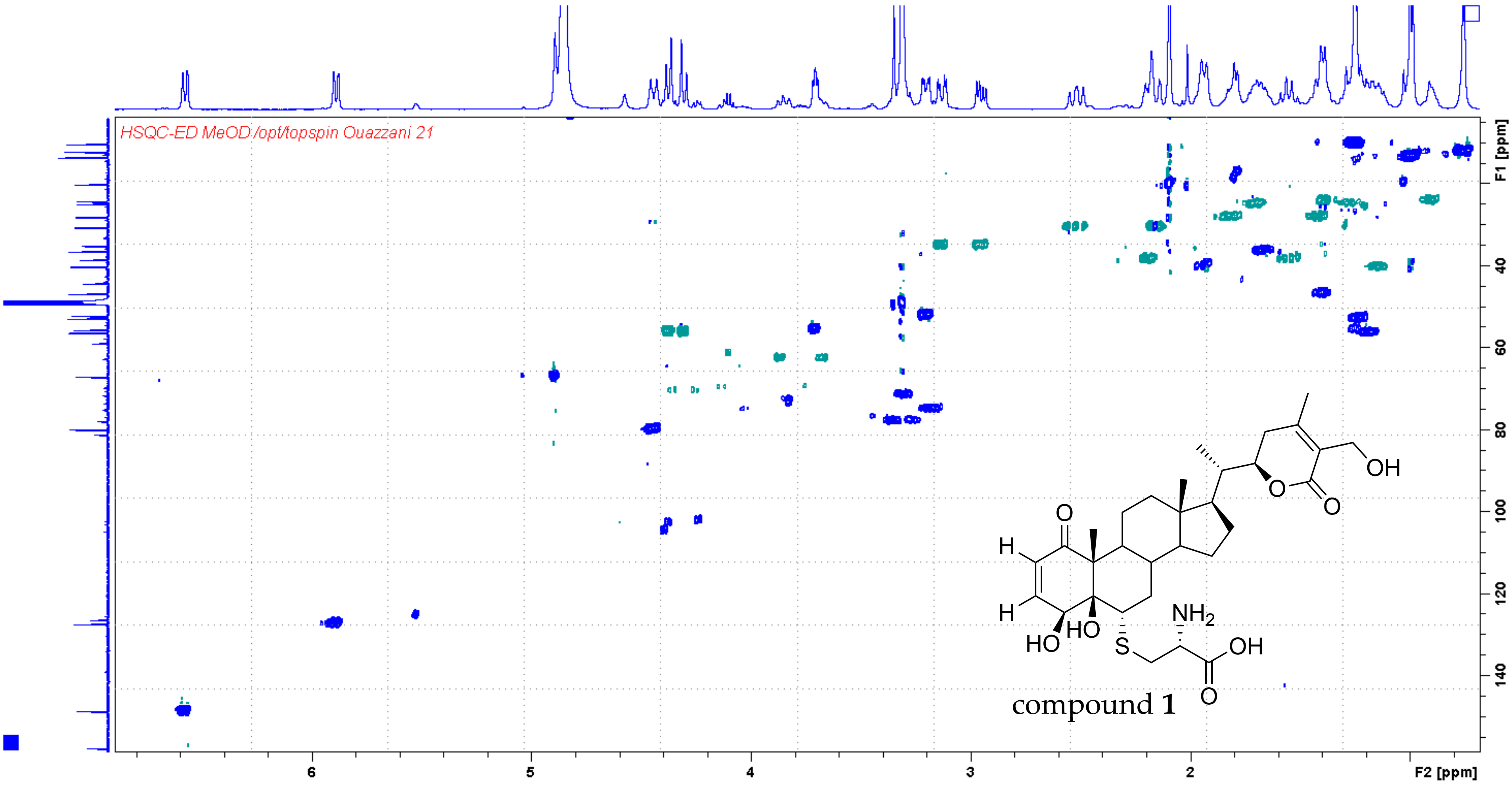

S4. ^{1}H-^{13}C HSQC-ED NMR spectrum (500 MHz, MeOD) of compound **1**

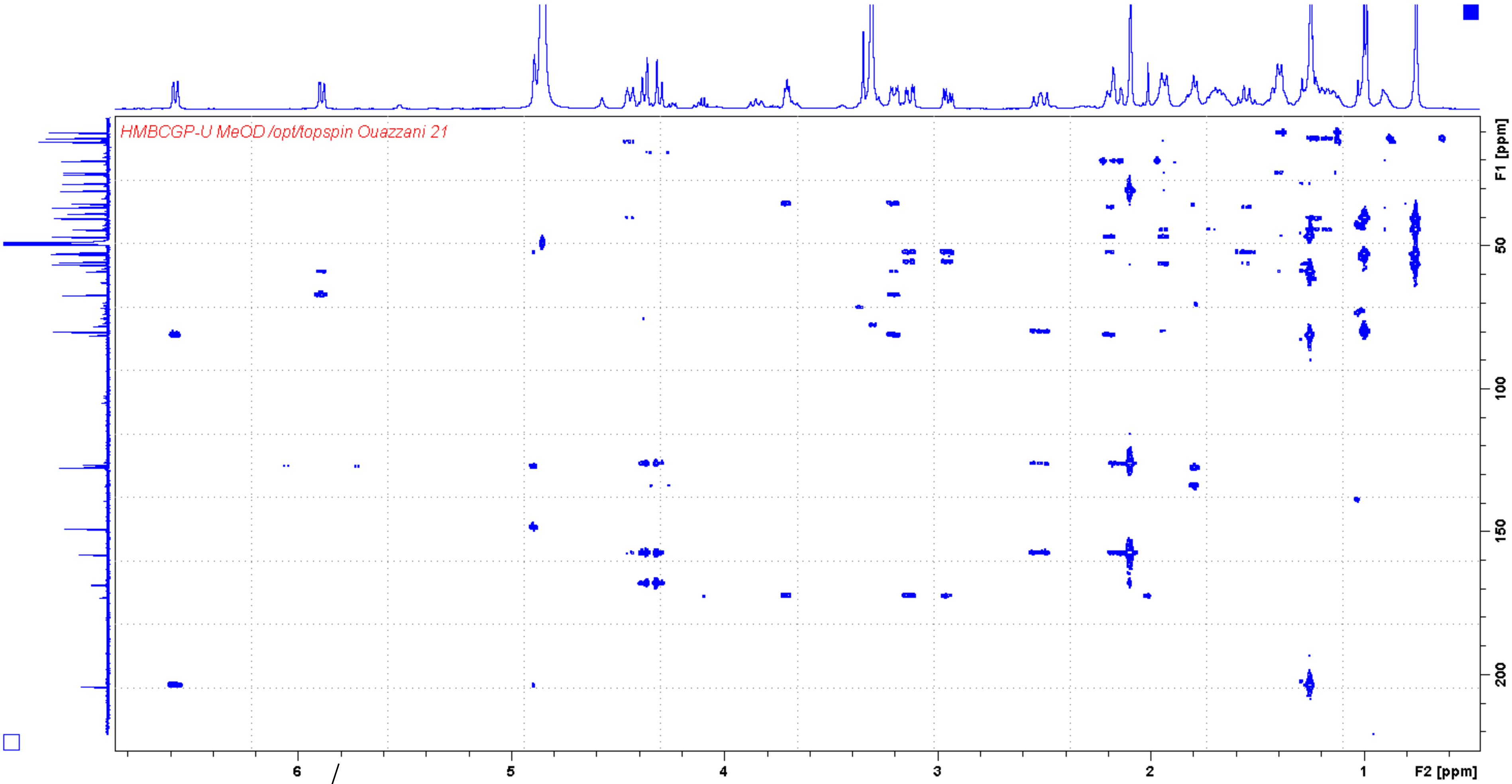

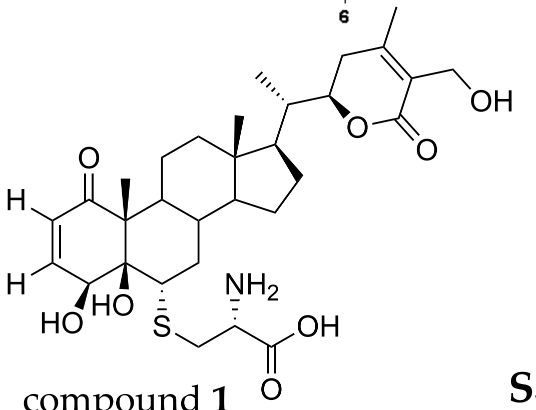

compound **1**

S5. ^{1}H-^{13}C HMBC NMR spectrum (500 MHz, MeOD) of compound **1**

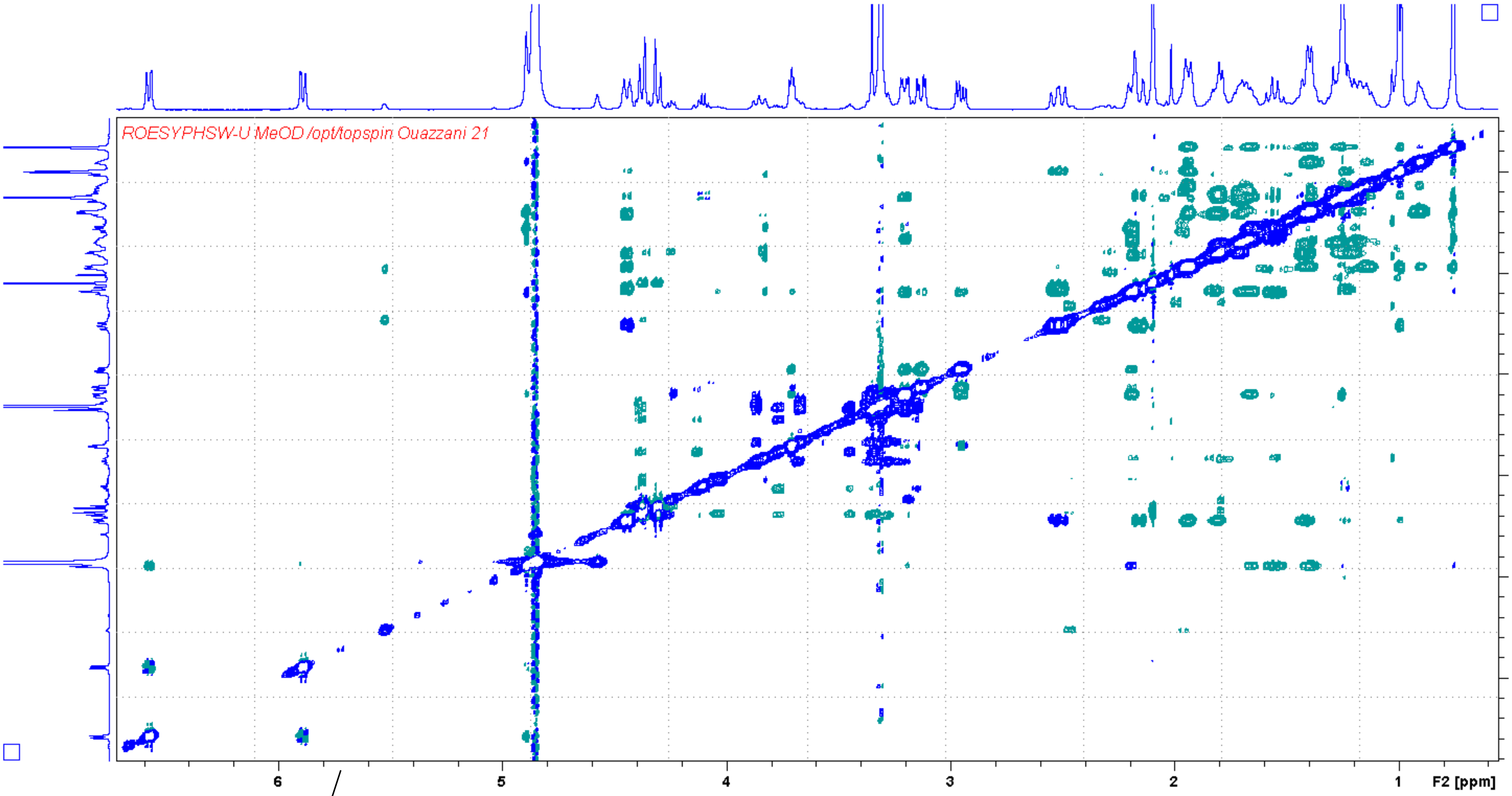

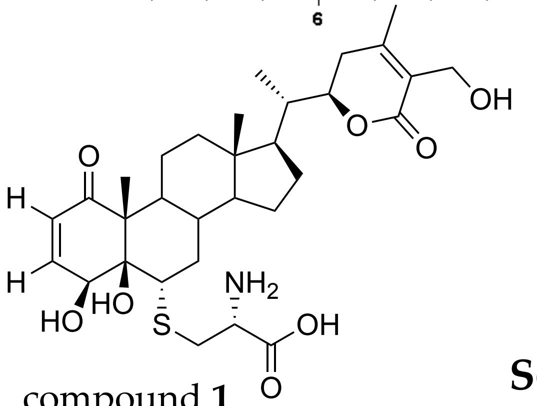

compound **1**

S6. ^{1}H-^{1}H ROESY NMR spectrum (500 MHz, MeOD) of compound **1**

Elemental Composition Report

Single Mass Analysis

Tolerance = 10.0 PPM / DBE: min = -1.5, max = 100.0
Element prediction: Off
Number of isotope peaks used for i-FIT = 9

Monoisotopic Mass, Even Electron Ions
1191 formula(e) evaluated with 9 results within limits (all results (up to 1000) for each mass)
Elements Used:
C: 0-50 H: 0-100 N: 0-3 O: 0-30 S: 0-1
OUAZZANI_arcile106-2 21 (0.573) Cm (16:26-37:68x2.000)

1: TOF MS ES+
1.63e+005

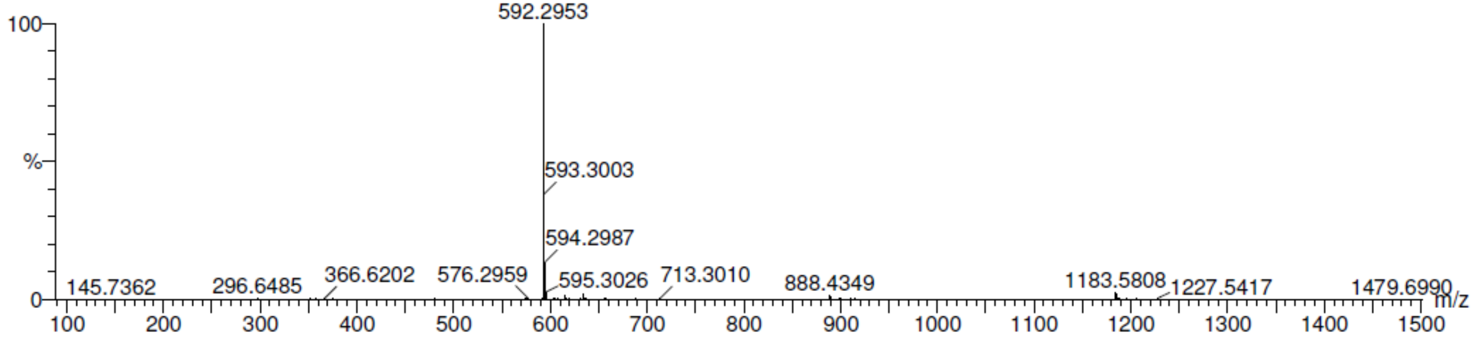

Minimum:				-1.5			
Maximum:		5.0	10.0	100.0			
Mass	Calc. Mass	mDa	PPM	DBE	i-FIT	i-FIT (Norm)	Formula
592.2953	592.2944	0.9	1.5	9.5	825.3	11.0	C31 H46 N O8 S
	592.2964	-1.1	-1.9	23.5	814.5	0.3	C40 H38 N3 O2
	592.2969	-1.6	-2.7	5.5	817.1	2.9	C27 H46 N O13
	592.2929	2.4	4.1	1.5	822.1	7.8	C22 H46 N3 O15
	592.2910	4.3	7.3	14.5	817.3	3.1	C34 H42 N O8
	592.2998	-4.5	-7.6	18.5	825.4	11.1	C37 H42 N3 O2 S
	592.2904	4.9	8.3	5.5	828.7	14.5	C26 H46 N3 O10 S
	592.3003	-5.0	-8.4	0.5	825.4	11.1	C24 H50 N O13 S
	592.3004	-5.1	-8.6	27.5	816.2	1.9	C45 H38 N

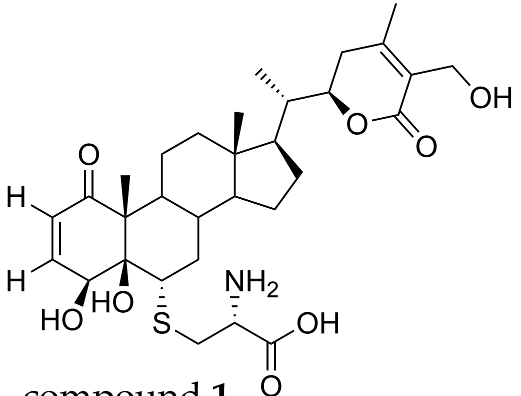

compound **1**

S7. HRMS spectrum of compound **1**

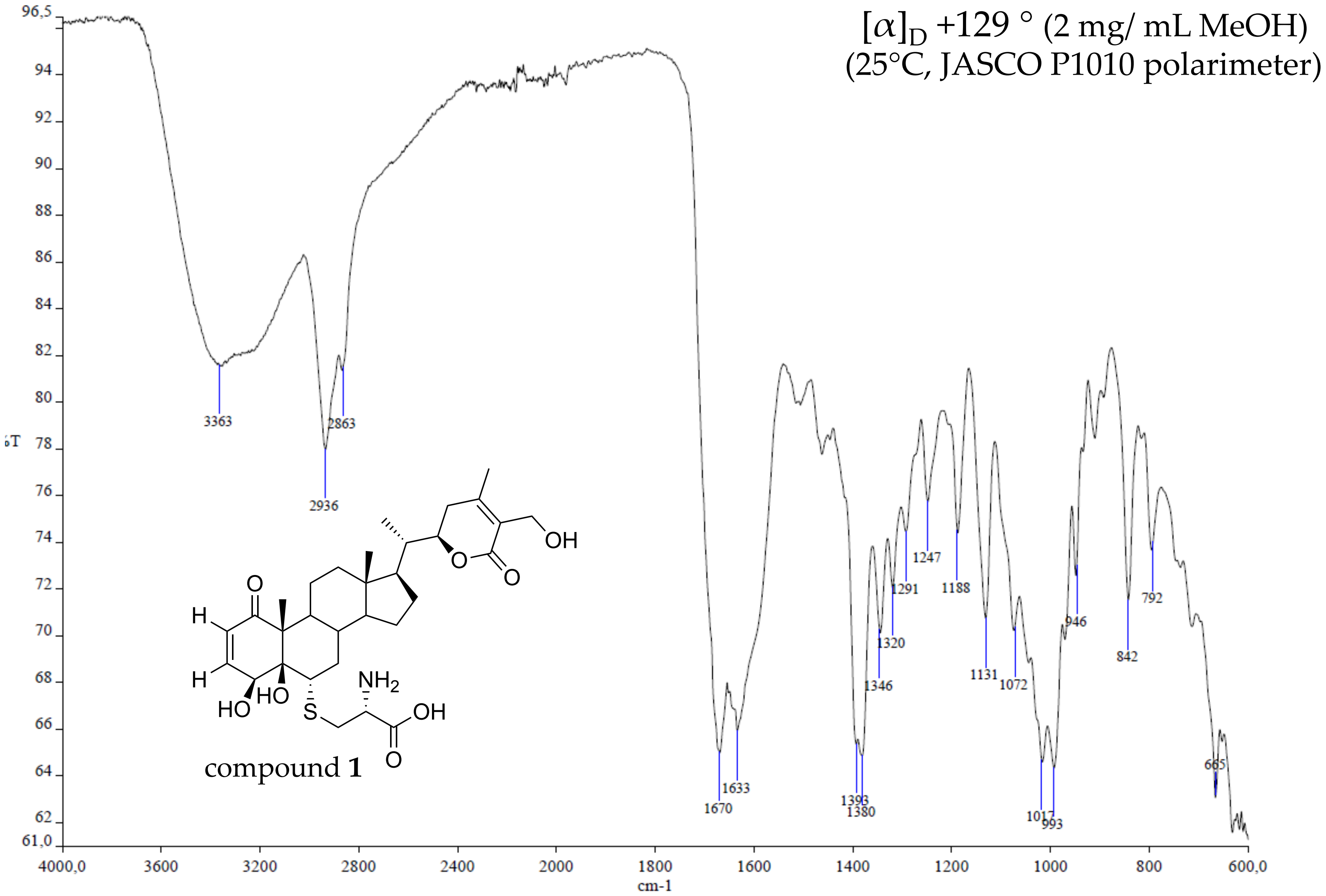

S8. IR spectrum of compound **1**

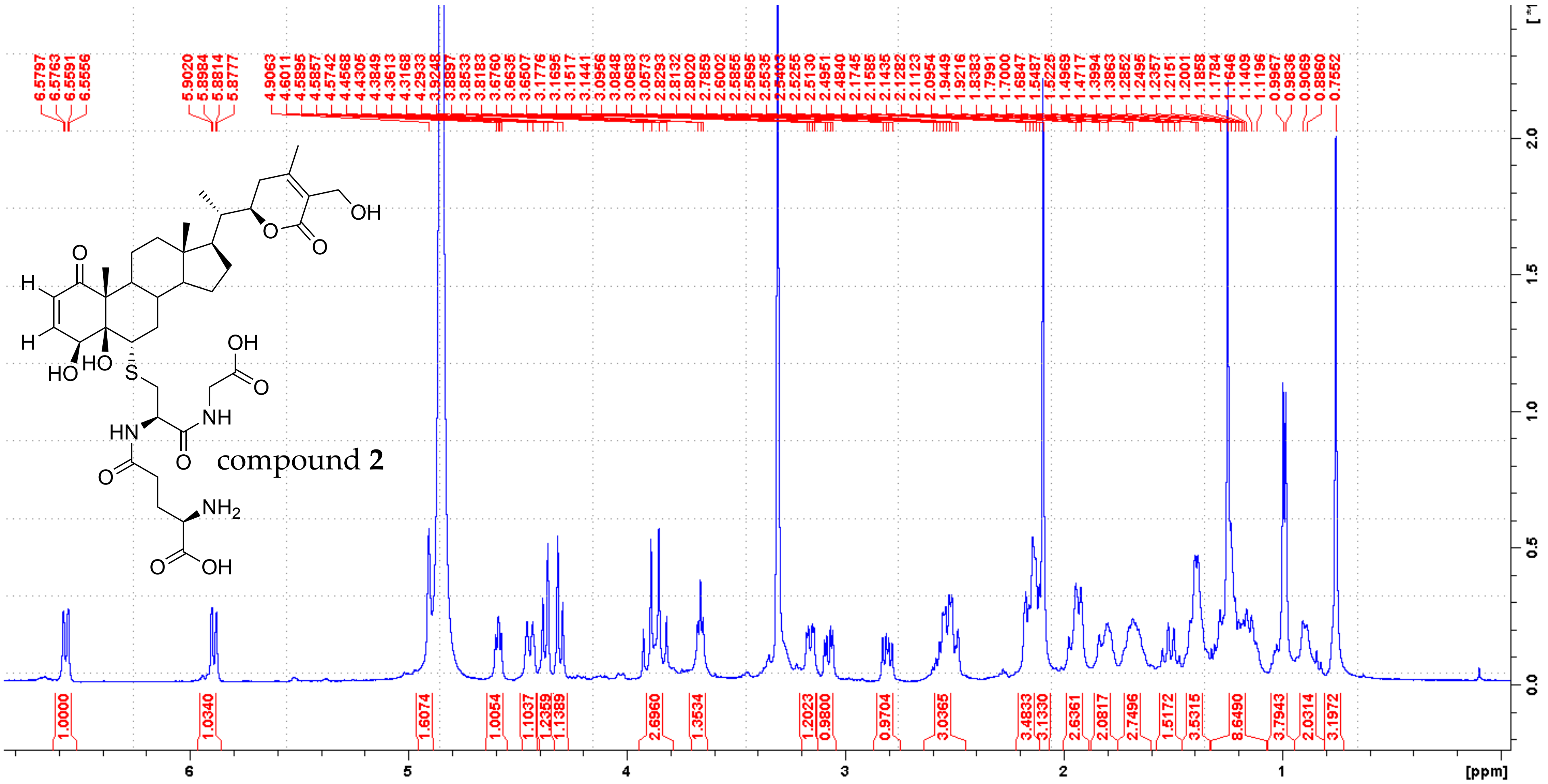

S9. ^{1}H-NMR spectrum (500 MHz, MeOD) of compound **2**

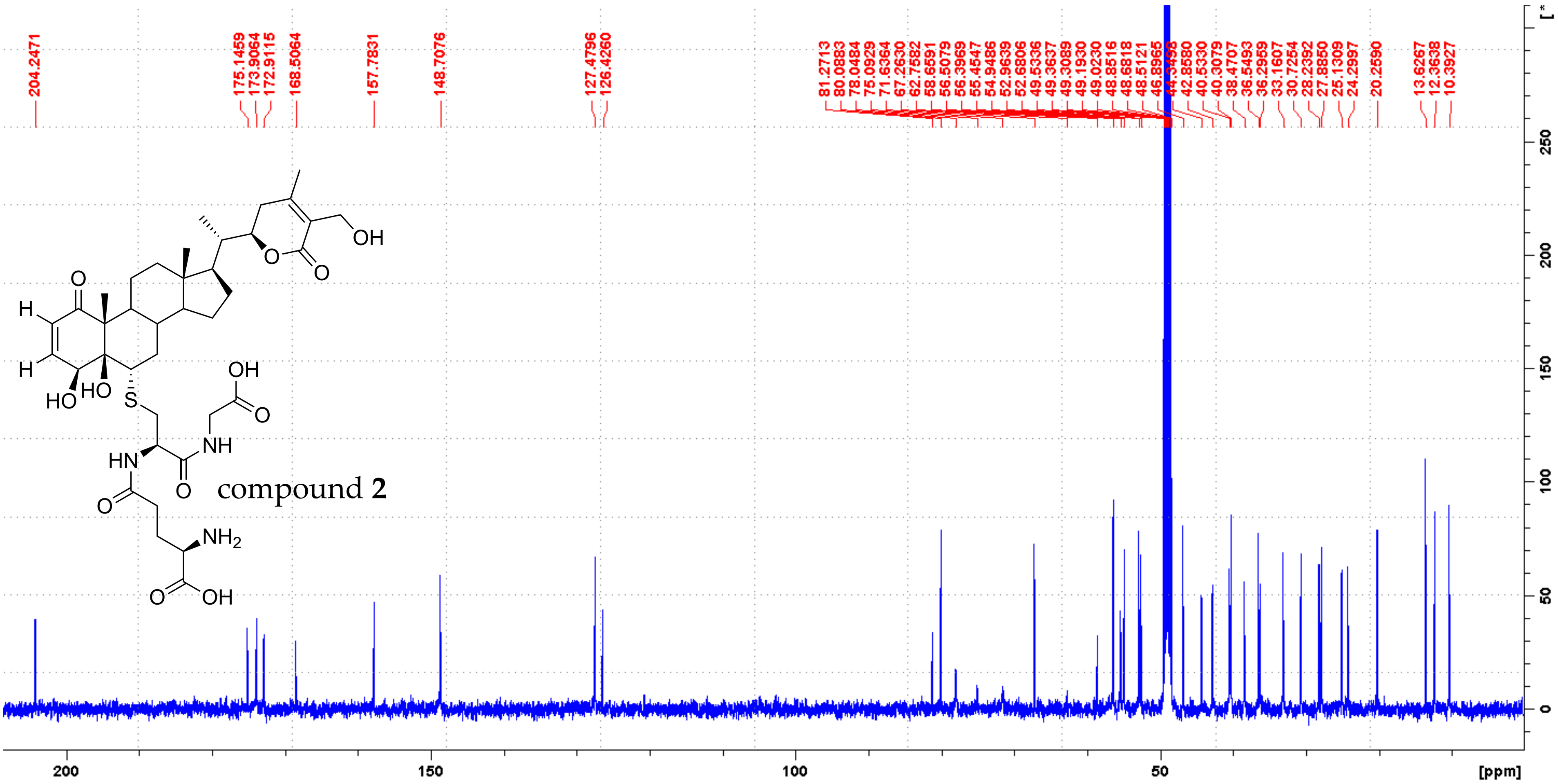

S10. ^{13}C-NMR spectrum (125 MHz, MeOD) of compound **2**

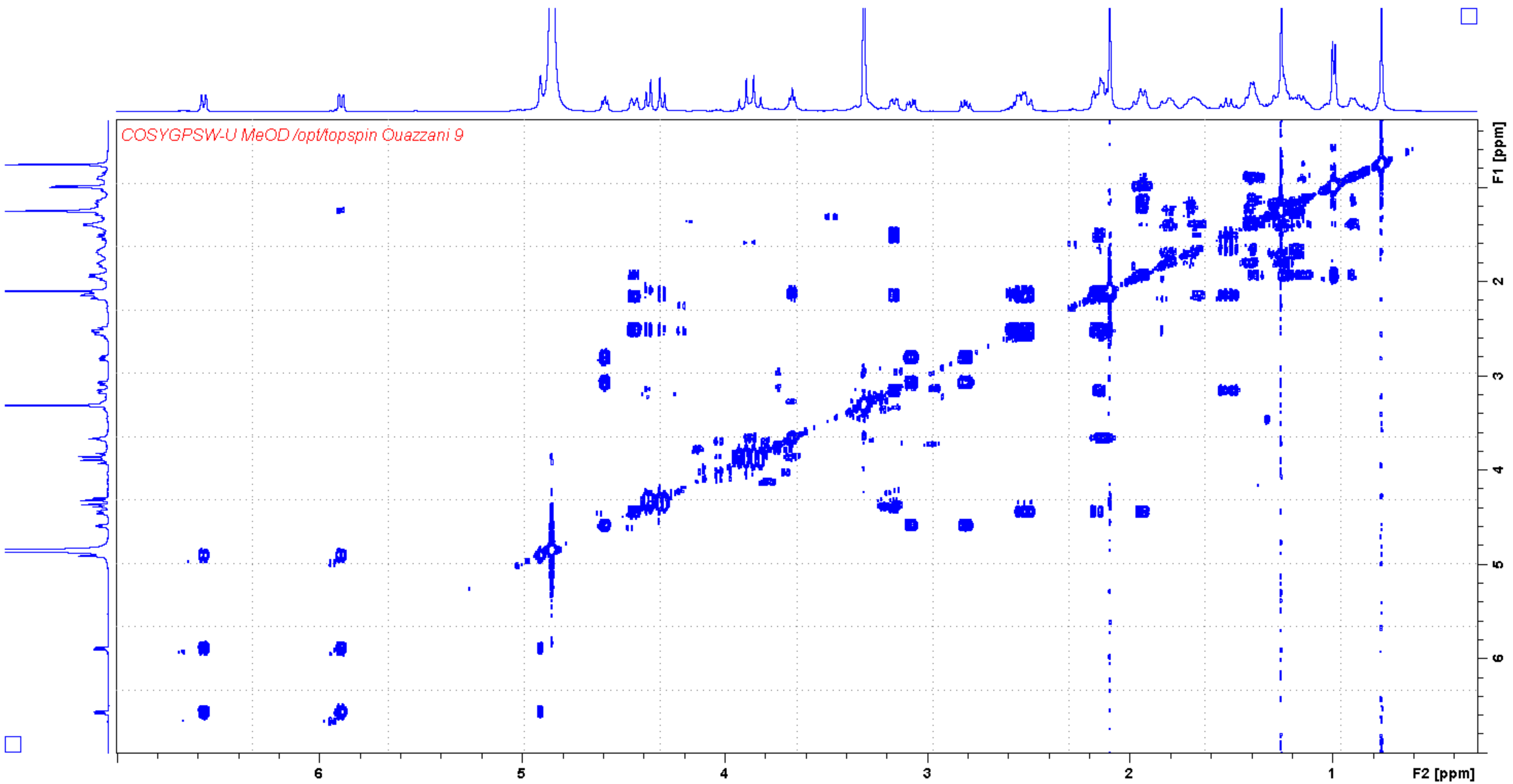

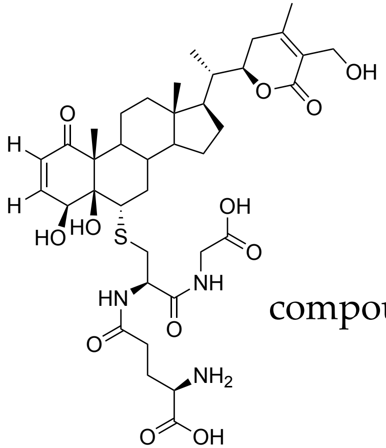

compound **2**

S11. ^{1}H-^{1}H COSY NMR spectrum (500 MHz, MeOD) of compound **2**

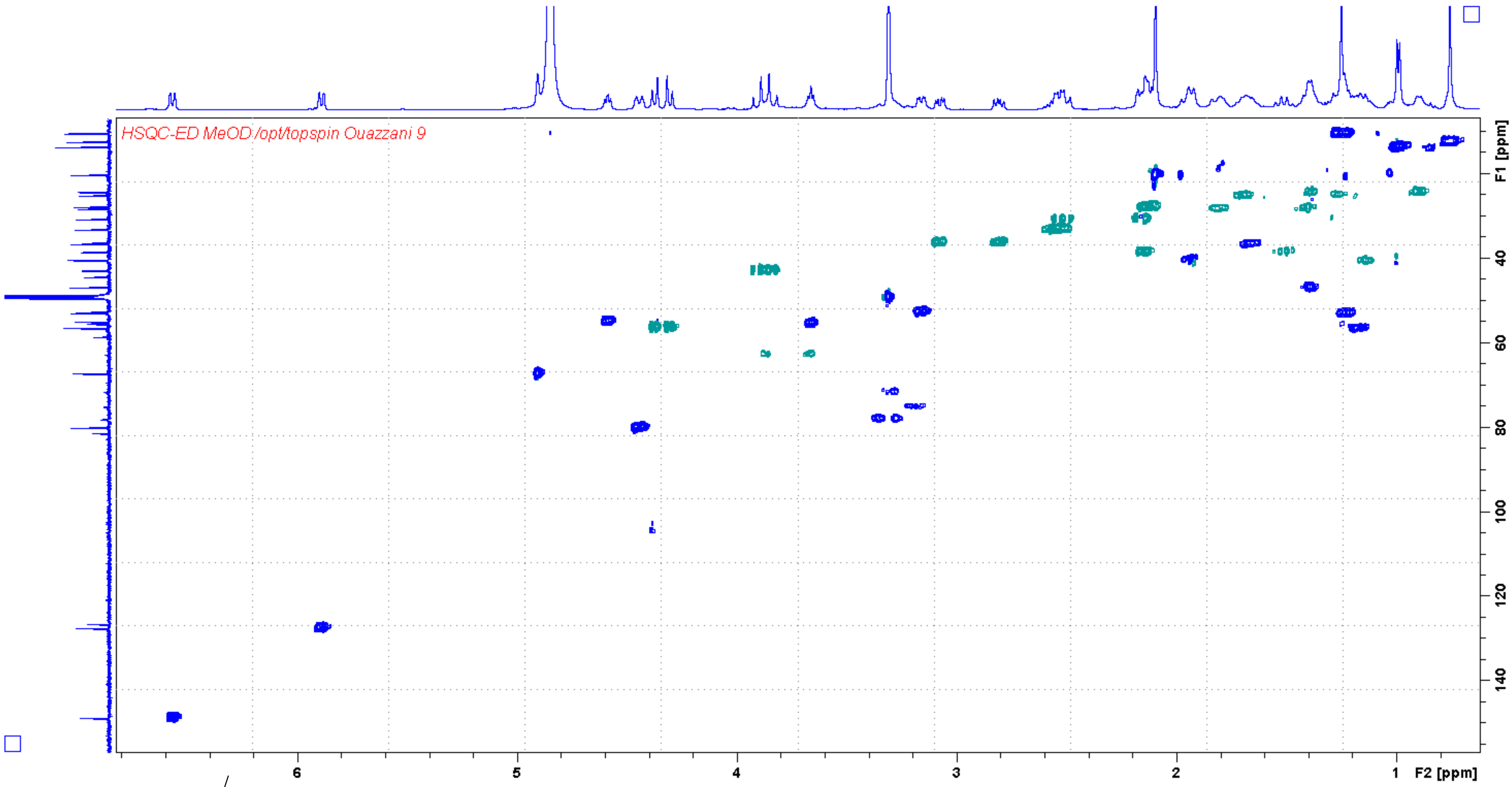

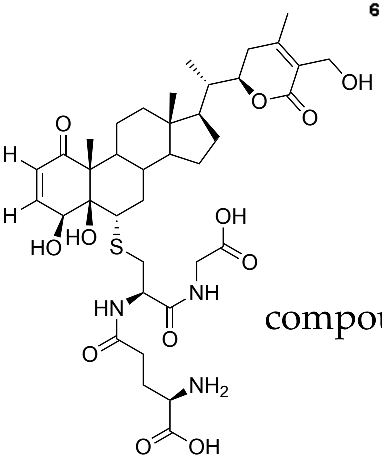

compound **2**

S12. ^{1}H-^{13}C HSQC-ED NMR spectrum (500 MHz, MeOD) of compound **2**

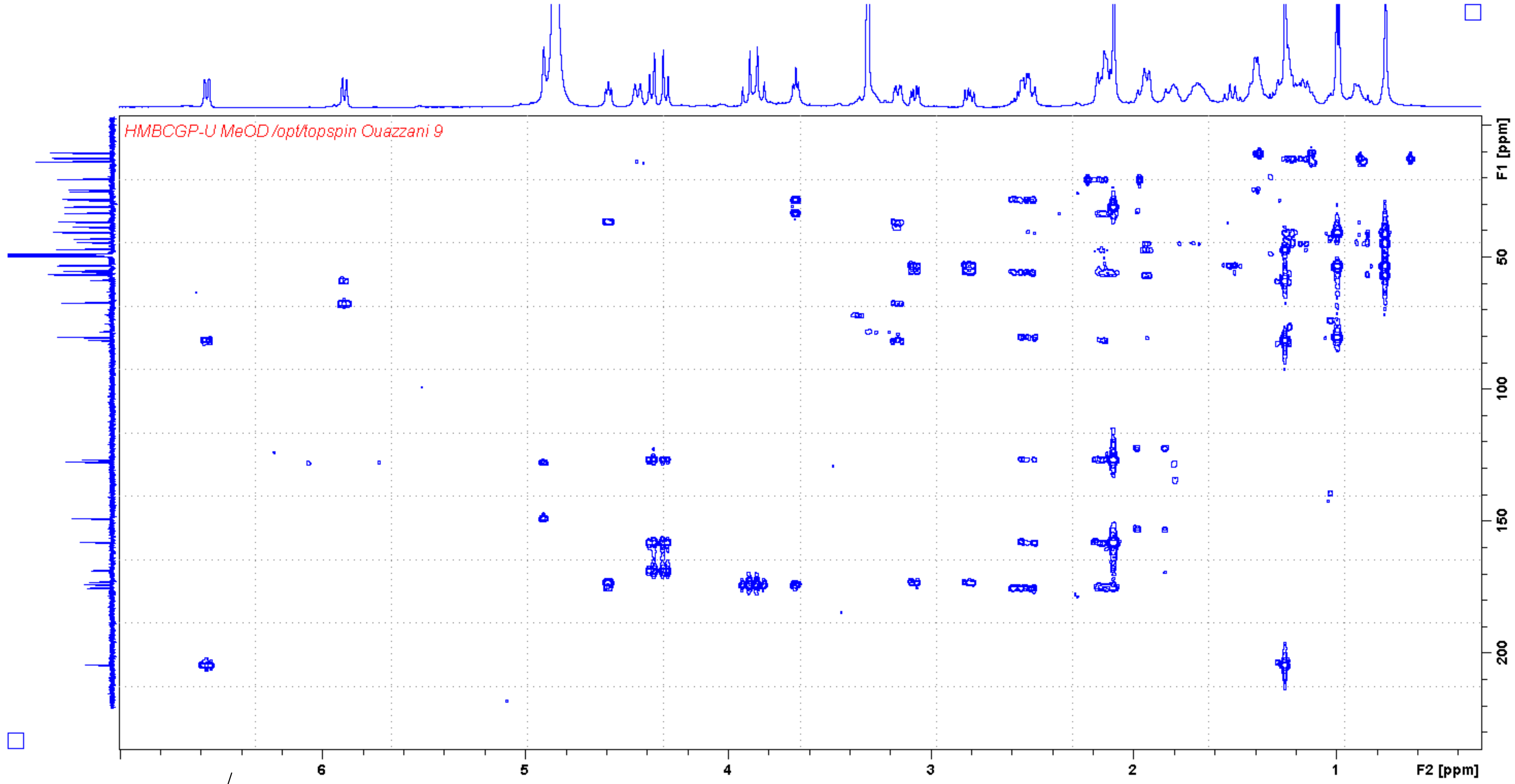

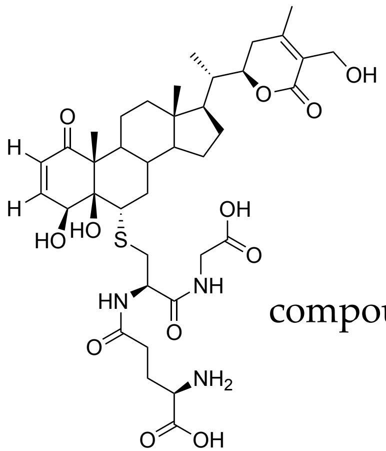

compound **2**

S13. ^{1}H-^{13}C HMBC NMR spectrum (500 MHz, MeOD) of compound **2**

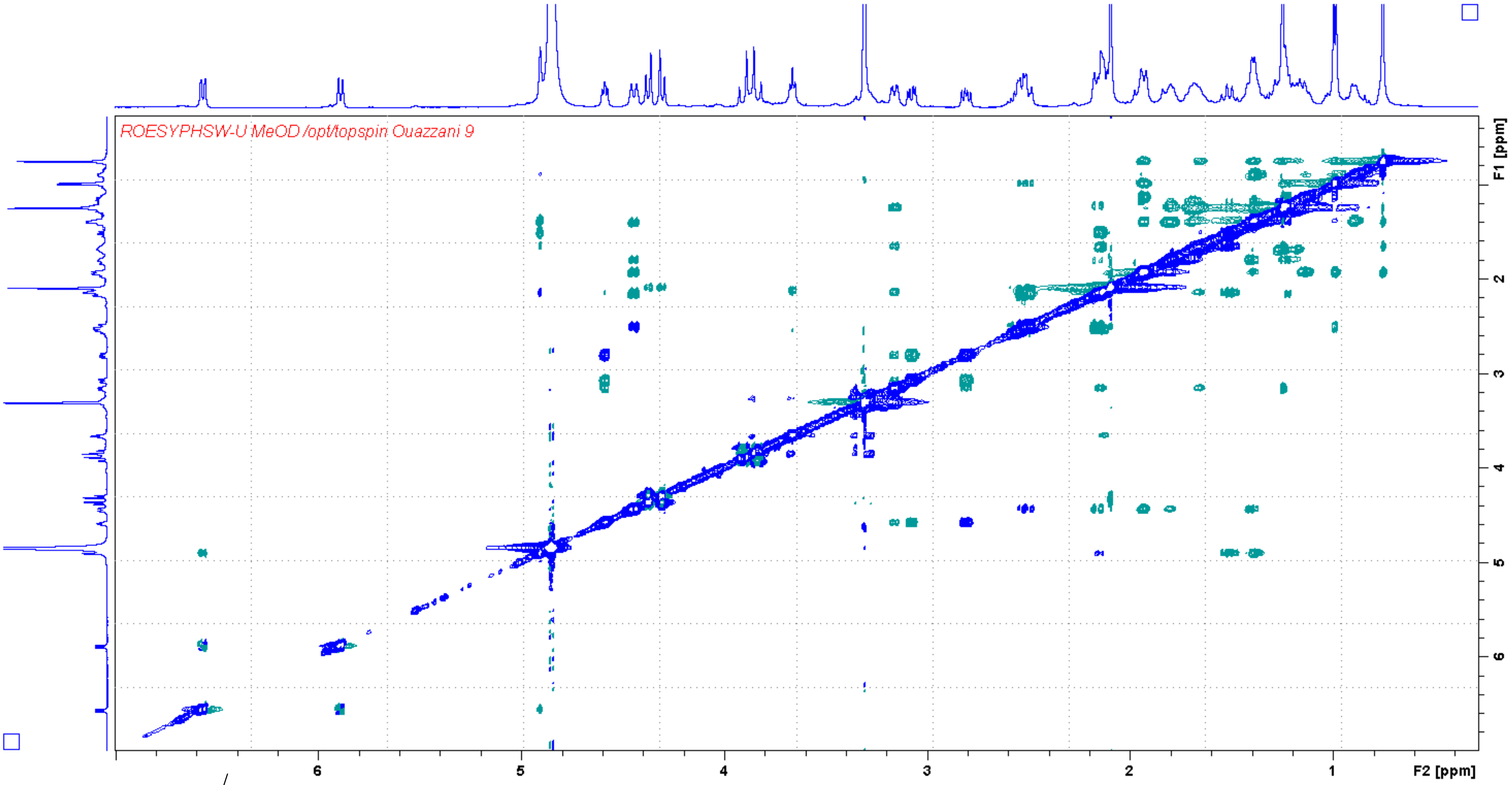

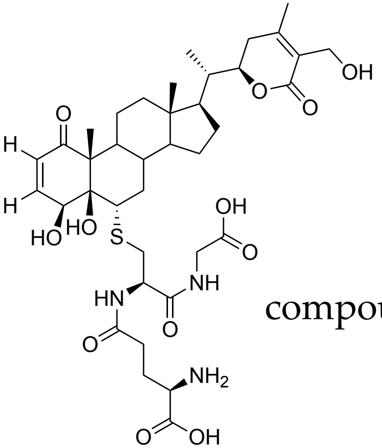

compound **2**

S14. ^{1}H-^{1}H ROESY NMR spectrum (500 MHz, MeOD) of compound **2**

Single Mass Analysis

Tolerance = 10.0 PPM / DBE: min = -1.5, max = 100.0
Element prediction: Off
Number of isotope peaks used for i-FIT = 9

Monoisotopic Mass, Even Electron Ions
240 formula(e) evaluated with 3 results within limits (all results (up to 1000) for each mass)
Elements Used:
C: 0-100 H: 0-100 N: 3-3 O: 0-100 S: 1-1
OUAZZANI_arcile64-1 24 (0.625) Cm (17:29-41:68x2.000)

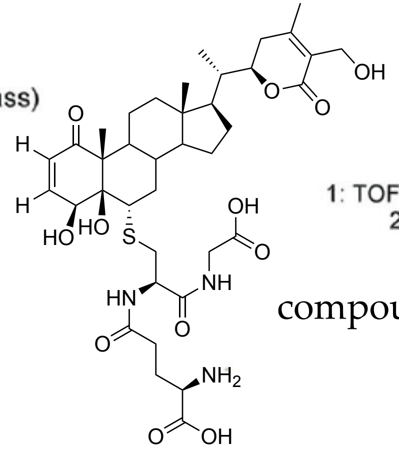

compound **2**

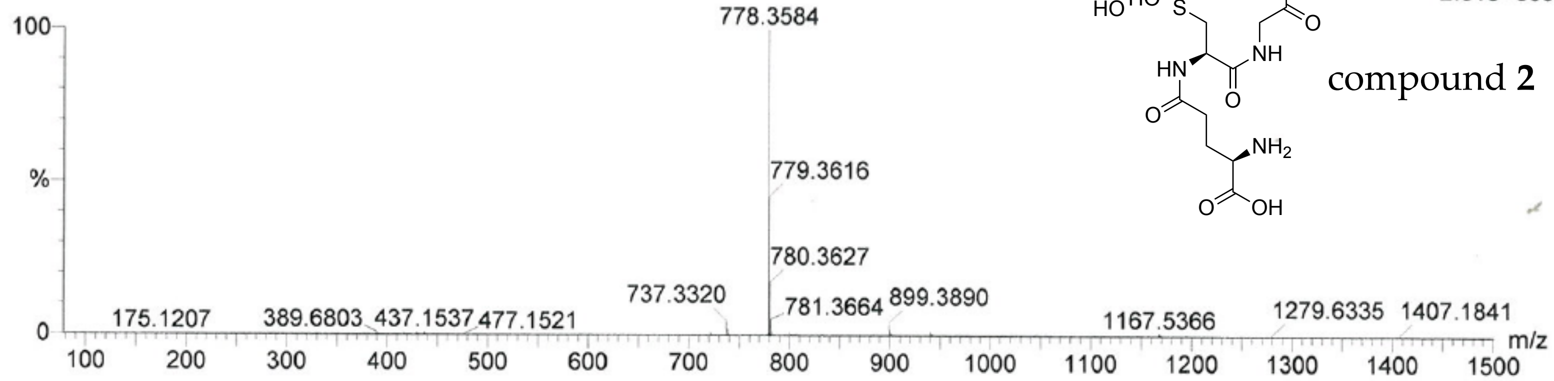

Minimum:				-1.5			
Maximum:		5.0	10.0	100.0			
Mass	Calc. Mass	mDa	PPM	DBE	i-FIT	i-FIT (Norm)	Formula
778.3584	778.3585	-0.1	-0.1	12.5	956.6	0.0	C38 H56 N3 O12 S
	778.3526	5.8	7.5	21.5	964.9	8.3	C45 H52 N3 O7 S
	778.3643	-5.9	-7.6	3.5	966.0	9.4	C31 H60 N3 O17 S

S15. HRMS spectrum of compound **2**

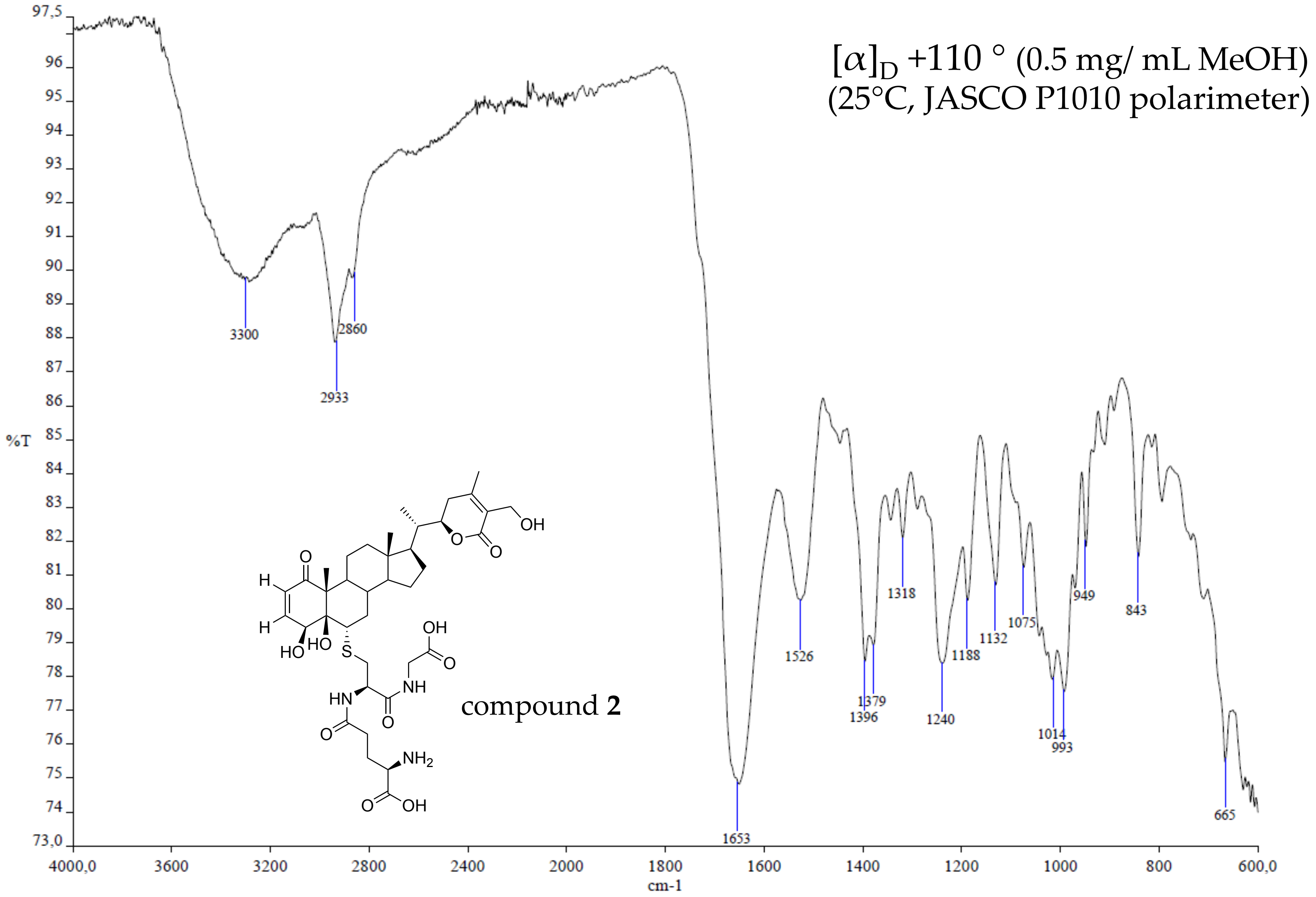

S16. IR spectrum of compound **2**
